# ﻿Seven new species of *Rinorea* (Violaceae) from the Neotropics

**DOI:** 10.3897/phytokeys.242.110474

**Published:** 2024-06-03

**Authors:** Saúl E. Hoyos-Gómez, Ricardo Callejas Posada, Gregory A. Wahlert

**Affiliations:** 1 Universidad de Antioquia, Instituto de Biología, Apartado postal 1226, Medellín, Colombia Universidad de Antioquia Medellín Colombia; 2 Cheadle Center for Biodiversity and Ecological Restoration, University of California, Santa Barbara, Santa Barbara, California, 93106, USA University of California Santa Barbara United States of America

**Keywords:** Conservation, Malpighiales, morphology, Neotropics, new species, taxonomy, Violaceae

## Abstract

Over the course of revising the genus *Rinorea* (Violaceae) from Colombia, field observations and herbarium studies revealed seven new species. Several of the new species described here belong to species complexes that required examination of herbarium material from across the Neotropics. Each of the new species described here have oppositely arranged leaves and belong to Rinoreasect.Pubiflorae, a section restricted to the Neotropics. Two new species are segregated from the *R.ovalifolia* species complex: *Rinoreachiribiquetensis* from Chiribiquete National Park in the Colombian Amazon and *Rinoreastevensii* from the Orinoco River near the border of Colombia and Venezuela. Two new species are segregated from the *Rinoreahirsuta* species complex: *Rinoreagaleanoae-bernalii* and *Rinoreacogolloi*, both from the eastern slopes of the Andean Central Cordillera along the mid-Magdalena River Valley in Colombia. From the widely distributed *R.pubiflora* species complex, we segregated one new species, *Rinoreacallejasii*, from southeast Panama and the Chocó in Colombia. In addition to these five new taxa segregated from widely distributed species complexes, we discovered two previously unknown species with affinities to other Neotropical *Rinorea*. *Rinoreaaymardii* is described from the Alto Orinoco-Casiquiare Biosphere Reserve in Venezuela and most closely resembles *R.melanodonta* from Colombia. *Rinoreabetancurii* is segregated from *R.macrocarpa* and occurs in the Amazonian Regions of Colombia, Brazil, Peru and Venezuela. In this study, we provide descriptions, illustrations and distribution maps of the new species and make preliminary assessments of the risk of extinction using the IUCN Red List Categories and Criteria. We also furnish an identification key to the species of Rinoreasect.Pubiflorae in Colombia.

## ﻿Introduction

The genus *Rinorea* Aubl. has a pantropical distribution and is the second largest genus in the Violaceae after *Viola* (Ballard et al. 2014). It is composed of 225–275 species of shrubs and trees with ca. 30 species in Asia, 115–155 species in Africa, 24 species in Madagascar and, in the Neotropics, where species of *Rinorea* typically occur in lowland rainforests, 47 species and two infraspecific taxa are currently recognised (Hekking 1988; Wahlert and Ballard 2009; Wahlert 2010; Marquete Ferreira da Silva and Medeiros 2012; Oliveira and de Queiroz 2020). They are often locally common shrubs and treelets of the forest understorey and frequently co-occur with other species of *Rinorea* (ter Stegee 2013).

In his taxonomic treatment of Neotropical *Rinorea*, Hekking (1988) classified the species into one of two groups: Super Group I ‘Apiculata’ and Super Group II ‘Rinorea.’ The three species in Super Group I ‘Apiculata’ were segregated into the genus *Bribria* Wahlert & H.E. Ballard, based on morphological, anatomical and molecular phylogenetic evidence (Wahlert et al. 2017; Wahlert and Ballard 2012; van Velzen et al. 2015). Hekking divided Super Group II ‘Rinorea’ into three groups: Group IIa ‘Rinorea,’ Group IIb, ‘Uxpanapana,’ and Group IIc ‘Pubiflora’ Hekking (1988). Wahlert et al. (2017) established these groups as taxa: Group IIa was recognised as Rinoreasect.Rinorea and includes all Neotropical *Rinorea* with alternately arranged leaves, Group IIb was segregated into the genus *Ixchelia* H.E. Ballard & Wahlert, with two species from Mexico and Mesoamerica and Group IIc was recognised as Rinoreasect.Pubiflorae Wahlert & H.E. Ballard and includes all Neotropical *Rinorea* with oppositely arranged leaves. The seven new species described in this study all belong to R.sectionPubiflorae, which is endemic to the Neotropics.

The present study was undertaken as part of a revision of *Rinorea* for Colombia. However, because several taxa are allied to widely distributed complexes, we examined specimens from across the Neotropics, from Mexico to Brazil and the Caribbean. We employed many of the same traits used by Hekking (1988) to circumscribe taxa and we found that previously overlooked characters of lamina venation are also useful in delimiting some species of *Rinorea*. The venation characters used in this study include details of the patterning of primary and tertiary veins and the spacing and angle of attachment of the secondary veins to the mid-vein, amongst others. In addition to three species discovered since Hekking’s (1988) monograph, the seven new species described here bring the total number of taxa in Rinoreasect.Pubiflorae to 44 (41 species and three infraspecific taxa). The 13 remaining Neotropical species, all with alternate leaves, belong to R.sect.Rinorea.

## ﻿Materials and methods

Field collections were made in lowland tropical rainforests in Bolivia, Colombia, Costa Rica and Peru, from 0–500 m elevation. All available herbarium material was examined from BHO, COAH, COL, F, FAUC, GH, HUA, JAUM, MEDEL, MO, NY, TOLI, UBDC and US (herbarium acronyms according to Thiers (2023)). Several digitised herbarium collections and records from the Global Biodiversity Information Facility (GBIF) were consulted to locate duplicate specimens for the following Herbaria: AAH, CM, FMB, G, INPA, K, MA, MBM, MG, MICH, RB, P, U and VEN. All cited collections have been seen by the first author. Species descriptions are based primarily on field observations and herbarium specimens. When available, flowers from herbarium specimens were rehydrated before dissection and measurement. A digital caliper and dissecting stereoscope were used to measure indument, flowers, fruits and seeds.

Leaf laminae were cleared to study venation architecture. Leaves were placed in a glass container and submerged in 1–5% sodium hydroxide (NaOH). The NaOH solution was changed every 1–2 days during the clearing process, which took 8–10 days. Leaves were then washed in a 50% Clorox^®^ solution followed by a final wash in water to stop the bleaching process. Staining was made with safranin for 5–8 minutes and then the leaves were put through a dehydration series in 50%, 95% and 100% ethanol (Ellis et al. 2009). The cleared leaves were photographed with a digital camera using a scale bar.

Post-factum georeferences for specimens lacking coordinates were assigned using either Google Earth or Tropicos specimen records with the same collecting localities. Collections lacking unambiguous locality information were omitted from the conservation assessment calculations. The online GeoCat facility (Bachman et al. 2011) was used to calculate the Extent of Occurrence (EOO) and Area of Occupancy (AOO) of each species to preliminarily estimate the risk of extinction using IUCN Red List Categories and Criteria (IUCN 2012); a 2 × 2 km cell was used for calculating AOO.

## ﻿Taxonomic treatment

### ﻿1. Rinorea callejasii

Taxon classificationPlantaeMalpighialesViolaceae

Hoyos-Gómez sp. nov.

31BA8214-4866-54EE-882C-AEA209E88248

urn:lsid:ipni.org:names:77342886-1

#### Type.

Panamá. Prov. Colón: forested slopes of Cerro El Jefe, near large coffee finca, 9°14'02"N, 79°22'30"W, 700–1000 m elev., 24 Jan 1970, *R. L. Wilbur et al. 11327* (holotype: MO [acc. 2027894; barcode MO-554811]!; isotypes: F [cat. 1694605]!, GH!, NY [barcode 04112199]!, US [cat. 2640360; barcode 03009254]!).

#### Description.

*Rinoreacallejasii* is most similar to R.pubifloravar.pubiflora s.s. by the lamina with symmetrical bases and bearing domatia, filaments free and fruit symmetrical, but it differs by the branchlets densely pubescent with ferruginous trichomes (vs. branchlets glabrescent in R.pubifloravar.pubiflora s.s.), primary and secondary veins on the abaxial lamina surface densely villous with ferruginous trichomes (vs. primary and secondary veins on the abaxial lamina surface glabrescent), fruit 1.5–2 cm long with 2 ovules per carpel (vs. fruit 2–3.5 cm long with 3 ovules per carpel) and the seeds glabrous (vs. seeds pubescent).

**Figure 1.**
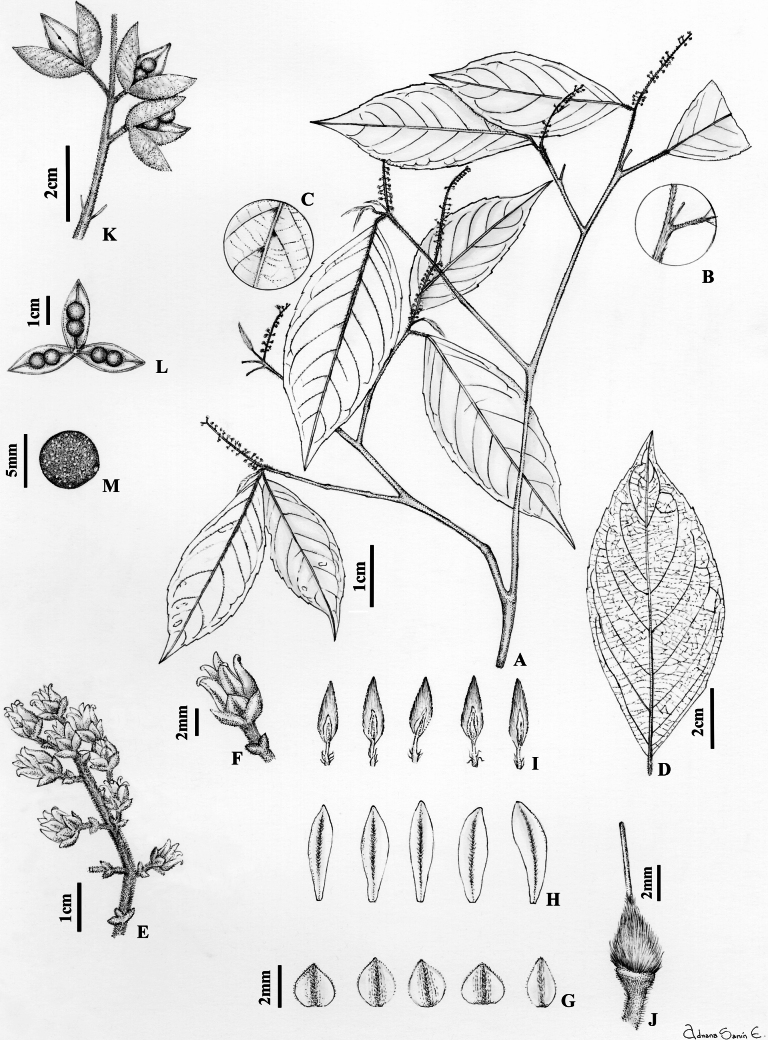
*Rinoreacallejasii* Hoyos-Gómez **A** habit **B** detail of young twig with pubescence **C** detail leaf showing domatia, abaxial surface **D** leaf architecture **E** inflorescence **F** flower **G** sepals, abaxial surface **H** petals, abaxial surface **I** stamens, adaxial surface **J** gynoecium **K** infructescence **L** fruit, showing two seeds per valve **M** seed. (**A–J**: *M. D. Correa 765* [MO]; **K–M**: *G. D. McPherson 6851* [MO]).

Shrubs or trees 2–12 m tall, terminal branchlets pubescent with erect ferruginous trichomes 0.4–0.5 mm long. Leaves opposite, petiolate; petioles 2–10 mm long, pubescent with erect ferruginous trichomes 0.2–0.3 mm long; stipules deciduous, free, elliptic, 1.6–4.8 × 0.8–1.3 mm, pubescent with appressed ferruginous trichomes 0.5 mm long; lamina elliptic, 4.6–15 × 2–6.5 cm, adaxial surface pilose on the mid-vein and secondary veins with erect ferruginous trichomes 0.4–0.5 mm long, abaxially pubescent on the mid-vein, trichomes on the secondary and tertiary veins, trichomes 0.3–0.5 mm long, erect, ferruginous, base cuneate, symmetrical, margin subserrate, apex acuminate, acumen 0.7–1.2 cm long, mucronate; with 8–10 major secondary vein pairs, major secondary veins eucamptodromous becoming brochidodromous distally, with regular spacing and uniform angle of the major secondary vein; major secondary attachment to mid-vein decurrent, leaf domatia present. Inflorescence axillary, terminal or subterminal, racemose, 3–5 cm long, 0.7–1.5 cm diam., axis pubescent with erect ferruginous trichomes 0.3–0.5 mm long; pedicels 1.8–3.6 mm long, pubescent with erect golden trichomes 0.3–0.5 mm long, articulated near the base; peduncle bracts deciduous, narrowly triangular, 1.5–1.8 × 1.5–1.8 mm, herbaceous, pubescent along the costa with appressed golden trichomes 0.3–0.5 mm long, margin ciliolate; bractlets persistent below articulation, subopposite, 1 × 1 mm. Flowers 3–3.6 × 3–3.4 mm; sepals subequal, triangular, 1.3–2 × 1.4–1.6 mm, 5- to 9-veined, pubescent along the costa with appressed golden trichomes 0.3 mm long, margin ciliolate, apex apiculate; petals lanceolate, 3.4–3.8 × 0.7–1.4 mm, pubescent along the costa with appressed golden trichomes 0.3–0.4 mm long, margin entire, apex acute, white; stamens 2.9–3.3 mm long, all filaments free, dorsal gland pilose with spreading golden trichomes 0.2–0.3 mm long covering lower half of the filament, anthers elliptic, 1.1–1.4 × 0.3–0.5 mm, apex rounded, connective 0.7–0.8 mm long, dorsal connective scale conspicuous, lateral as well as apical, lanceolate, 2.2–2.5 × 0.8–1 mm, margin entire, orange-brown; ovary globose, 0.8–1 × 0.9–1 mm, pubescent with erect, golden trichomes 0.4–0.5 mm long; style erect, filiform, 2–2.3 × ca. 0.1 mm, pubescent proximally with appressed golden trichomes 0.2–0.3 mm long, stigma undifferentiated. Fruit a symmetrical, subligneous capsule dehiscent along three sutures, ellipsoid, 1.5–2 × 0.5–0.6 cm, apex acuminate, pubescent with erect golden trichomes 0.2–0.3 mm long, green in vivo, brown when dry. Seeds two per valve, globose, 4–5 mm diam., glabrous, with maculae, brown when dry. (Fig. 1)

#### Distribution and habitat.

**Figure 2.**
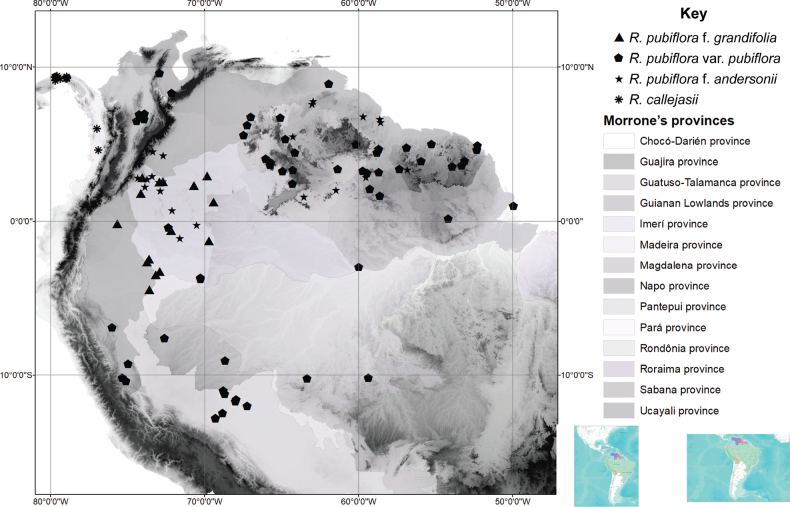
Distribution of R.pubifloraf.grandifolia (triangles), R.pubifloravar.pubiflora (pentagons), R.pubifloraf.andersonii (stars), *Rinoreacallejasii* (asterisks).

*Rinoreacallejasii* is restricted to the Caribbean coast of Colón and San Blas Provinces, Panama and the Pacific coast of Chocó Department, Colombia, a region belonging to the biogeographic Province of Chocó-Darien in the Pacific Dominion (*sensu*Morrone (2014)). It occurs in lowland humid rainforests at elevations of 30–500 m. (Fig. 2)

#### Etymology.

*Rinoreacallejasii* honours Dr. Ricardo Callejas Posada, Professor of Biology at Universidad de Antioquia, Medellín, Colombia. Prof. Callejas has made many contributions to the taxonomic knowledge of the Piperaceae and expanded the understanding of the diverse flora of Colombia, especially in the Department of Antioquia. He was the major advisor of SEHG.

#### Phenology.

The species flowers January through March; specimens in fruit were collected in March and May.

#### Conservation status.

*Rinoreacallejasii* has a geographic range in the form of an EOO of 40,729 km^2^ and AOO of 52 km^2^. In Panama, it is represented by two locations within Chagres National Park and one location in Soberanía National Park. Outside of the protected areas, where the species exists at nine locations, it is threatened by the exploitation of natural resources, mineral extraction and energy development. In Colombia, it is represented by two locations, both outside protected areas. The species does not meet the criteria necessary to assign a threatened status. However, given ongoing habitat disturbance, it is likely that the species will qualify in the near future for a threatened status and, thus, it is preliminarily assessed with a status of “Near Threatened” (NT).

#### Notes.

*Rinoreapubiflora*, as circumscribed by Hekking (1988), was morphologically variable across its large distribution in the Neotropics. He recognised a variety of *R.pubiflora*: var.grandifolia (Eichler) Hekking, which was further divided into two forms: f.grandifolia and f.andersonii (Sandwith ex Hekking) Hekking. *Rinoreacallejasii* is a segregate of R.pubifloravar.pubiflora s.s. In attempting to explain the morphologically divergent specimens of var. pubiflora from Panama (e.g. *G. C. de Nevers et al. 5075*), Hekking (1988) invoked a scenario of hybridisation between R.squamataS.F.Blake andvar.pubiflora. However, our analysis of morphological divergence and biogeographic distribution suggest that *R.callejasii* is a separately evolving lineage from the nominate species (*sensu*De Queiroz (2008)) and deserving of recognition as a new taxon at the rank of species.

*Rinoreacallejasii* is sympatric with four other taxa of *Rinorea*. *Rinoreasquamata* and *R.hirsuta* Hekking can be differentiated by their single glabrous seed per valve (vs. two glabrous seeds per valve in *R.callejasii*). *Rinoreadasyadena* is distinguished by its two pubescent seeds per valve and asymmetrical lamina base (vs. two glabrous seeds and symmetrical lamina base). Rinorealindenianavar.fernandeziana can be separated by its one pubescent seed per valve (vs. two glabrous seeds per valve).

#### Additional specimens examined.

**Colombia. Dept. Chocó**. Mpio. de San José del Palmar: Hoya del río San Juan, 04°36'N, 76°54'W, 7 Apr 1979 (fr), *E. Forero et al. 4780* (COL, MO, US); Mpio. Bahía Solano: hills behind Bahía Solano, 6°13'14"N, 77°24'27"W, 0–250 m elev., 5 Jan 1973 (fl), *A. H. Gentry & E. Forero 7245* (COL, F, MO, U). **Panamá. Prov. Colón**: Santa Rita Ridge lumber road, 23 Feb 1968 (fl), *M. D. Correa-A. & R. L. Dressler 765* (MO, NY, US); Santa Rita Ridge, 9°19'N, 79°39'W–9°24'N, 79°48'W, 1 Mar 1971 (fl), *T. B. Croat 13831* (MO); El Llano-Cartí Road, 10 miles from Inter-American Highway near El Llano, 9°17'45"N, 78°56'15"W, 330 m elev., 28 Mar 1976 (fl, fr), *T. B. Croat 33754* (COL, MO); El Llano-Cartí Road, 5–6 miles N of Inter-American Highway at El Llano, 09°15'30"N, 78°55'50"W, 350–375 m elev., 7 May 1976 (fl), *T. B. Croat 34788* (COL, MO); El Llano-Cartí Road, km 26.5, trail NE from road, [coordinates on original label: 9°19'N, 78°55'W; corrected to 9°22'N, 78°58'W], 175 m elev., 9 Mar 1985 (fl), *G. C. de Nevers et al. 5075* (MBM, MO); Santa Rita Ridge road, 4 miles from Transisthmian Highway to Agua Clara weather station, 9°21'N, 79°42'W, 11 Dec 1973 (fl), *R. L. Dressler et al. 8846* (MO); Santa Rita Ridge road, 4 miles from Transisthmian Highway to Agua Clara weather station, 9°21'N, 79°42'W, 500 m elev., 11 Dec 1973 (fl), *A. H. Gentry et al. 8846* (MO, COL); along road into Santa Rita, E of Agua Clara, 9°07'10"N, 79°42'02"W, 4 Mar 1973 (fl), *H. Kennedy 2741* (MO); Santa Rita Ridge, 9°20'N, 79°45'W, 13 May 1986 (fl), *G. D. McPherson 9168* (MG, MO); 12.7 km from Inter-American Highway, 350 m elev., 15 Feb 1975 (fr), *S. A. Mori 4694* (COL); Santa Rita Ridge Road, 17 km from Boyd-Roosevelt Hwy, 9°22'N, 79°40'W, 450 m elev., 14 Mar 1975 (fl), *S. A. Mori et al. 5041* (COL, MO); El Llano-Cartí Road, 20.7 km north from Inter-American Hwy, 9°17'58"N, 78°55'58"W, 20 Mar 1975, S. A. Mori & J. A. Kallunki 5115 (COL, MO); 9 km from Inter-American Highway, 9°15'50"N, 78°55'51"W, 350 m eelv, 22 Mar 1975 (fr), *S. A. Mori 5154* (COL, MO); upper Río Piedras headwaters, along trail from end to Santa Rita Ridge road, ca. 11 km SW of Cerro Braja, 9°25'N, 79°35'W, 4 May 1981 (fr), *K. J. Sytsma et al. 4311* (MO). **Prov. San Blas**: El Llano-Cartí Road, 7 miles on Interamerican Highway, 9°15'N, 79°00'W, 550 m elev., 14 Mar 1985 (fr), *G. D. McPherson 6851* (MO).

### ﻿2. Rinorea aymardii

Taxon classificationPlantaeMalpighialesViolaceae

Hoyos-Gómez sp. nov.

BAA35902-1C66-5E2B-A544-1177489AD134

urn:lsid:ipni.org:names:77342872-1

#### Type.

Venezuela. Dept. Amazonas de Atabapo: “Caño Iguapo” Alto Orinoco, 15 km al SE de la Esmeralda, 3°8'N, 65°27'W, 100–180 m elev., 20 Feb 1990, *G. Aymard et al. 8050* (holotype: MO [acc. 5983541; barcode MO-1980109]!; isotype: MA [cat. 543845]!).

#### Description.

*Rinoreaaymardii* is similar to *R.melanodonta* S.F.Blake by the lamina with domatia and symmetrical bases, filaments free and style erect, but it differs by the smaller, herbaceous, lanceolate lamina, 4–11 × 1–2.7 cm (vs. larger, coriaceous, elliptic lamina, 6.5–17 × 1.7–5.2 cm in *R.melanodonta*), 7–8 major secondary vein pairs (vs. 9–12 major secondary vein pairs), abaxial tertiary venation percurrent, pubescent (vs. abaxial tertiary veins reticulate, glabrescent), petals 2.8–3 × 1–1.2 mm, with entire margin and pubescent at the apex (vs. petals 5–5.7 × 1.5–2.2 mm, with margin ciliolate and glabrous at the apex), shorter stamens, 2.8–3 mm long (vs. longer stamens, 4–4.5 mm long), dorsal gland glabrous (vs. dorsal gland pilosulous) and smaller anther connective scales, 2–2.1 × 0.7–0.8 mm (vs. larger anther connective scales, 3.2–3.5 × ca. 1.5 mm).

**Figure 3.**
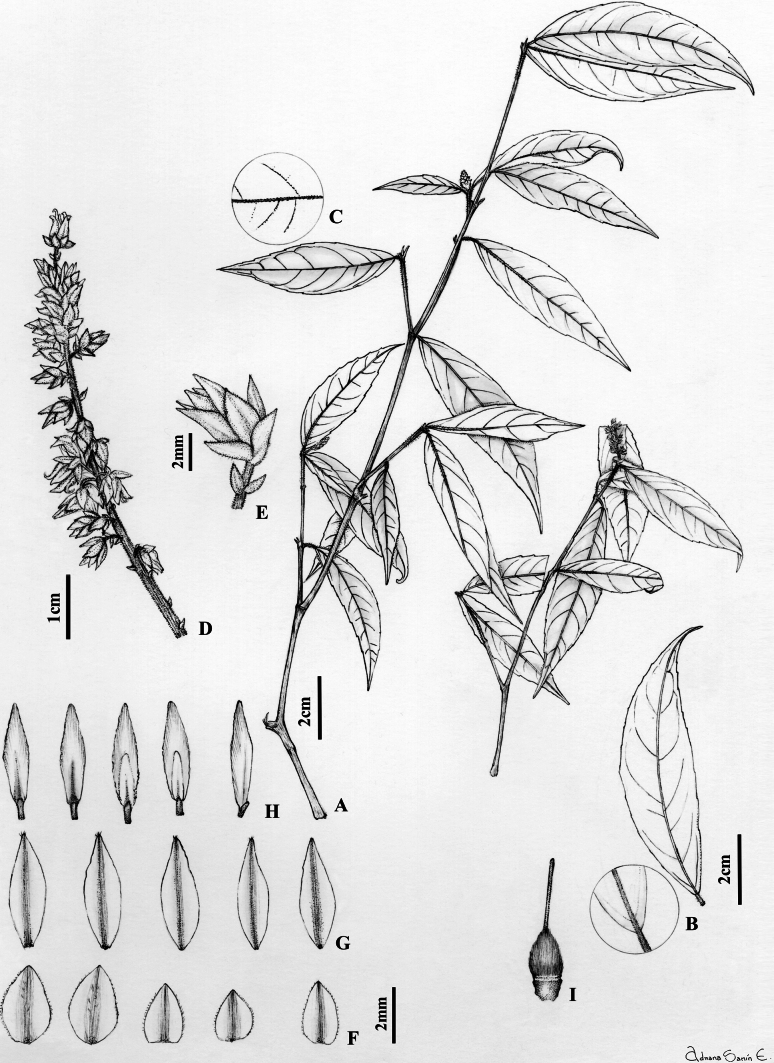
*Rinoreaaymardii* Hoyos-Gómez **A** habit **B** leaf architecture **C** detail of leaf, abaxial surface **D** infructescence **E** flower **F** sepals, abaxial surface **G** petals, abaxial surface **H** stamens, showing both surfaces **I** gynoecium. (**A–I**: *G. Aymard 8050* [MO]).

Treelets to 3 m tall, young branchlets strigose with erect golden trichomes 0.1–0.2 mm long, glabrescent. Leaves opposite, petiolate; petiole 1.8–3.8 mm long, pubescent with erect golden trichomes 0.1–0.2 mm long; stipules deciduous, free, lanceolate 2–2.4 × 0.9–1 mm, herbaceous, pubescent with appressed golden trichomes 0.1–0.2 mm long, apex mucronate; lamina elliptic-lanceolate, 4–11 × 1–2.7 cm long, adaxially glabrous, abaxially pubescent on the mid-vein and veins with appressed golden trichomes 0.2–0.4 mm long, with 7–8 major secondary vein pairs, secondary veins with irregular spacing, vein angles smoothly decreasing proximally, mixed epimedial tertiary veins, symmetrical and rounded base, margin crenate, apex acuminate, acumen 0.5–1 cm, mucronate, leaf domatia absent. Inflorescence axillary, terminal or subterminal, racemose, 1.7–4 cm long, 0.8–1 cm diam., axis pubescent with erect golden trichomes 0.2 mm long; pedicels 1.7–2 mm, pubescent with erect golden trichomes 0.2 mm long, articulated near the middle; bractlets persistent below articulation, opposite, ca. 1 × 1 mm, herbaceous, the costa pubescent with appressed golden trichomes 0.2 mm long, peduncle bracts triangular, ca. 1 × 1.2 mm, herbaceous, pubescent with appressed ferruginous trichomes 0.2 mm long, margin ciliolate. Flowers 3–3.5 × 3–3.5 mm, sepals subequal in size and shape, triangular, 1.4–2.4 × 1–1.3 mm, 3–5-veined, costa pubescent with appressed golden trichomes, 0.2 mm long, margin ciliolate, apex apiculate; petals lanceolate, 2.8–3 × 1–1.2 mm, pubescent with erect golden trichomes 0.1–0.2 mm long near the apex, margin entire, apex acute, cream-coloured in vivo, brown when dry; stamens 2.8–3 mm long, filaments free, glabrous, 0.4–0.6 mm long, dorsal glands glabrous, completely covering the filaments; anthers lanceolate, 1–1.2 × 0.4–0.5 mm, glabrous, apex rounded, connective 1–1.2 mm long, dorsal connective scale lanceolate, 2–2.1 × 0.7–0.8 mm, scarious, brown–orange, margin subentire; ovary globose, 0.8–1 × 0.8–0.9 mm, pubescent with erect golden trichomes 0.2–0.3 mm long; style erect, filiform, glabrous, 1.7–1.9 × ca. 0.1 mm, stigma acute. Fruit unknown. (Fig. 3)

#### Distribution and habitat.

**Figure 4.**
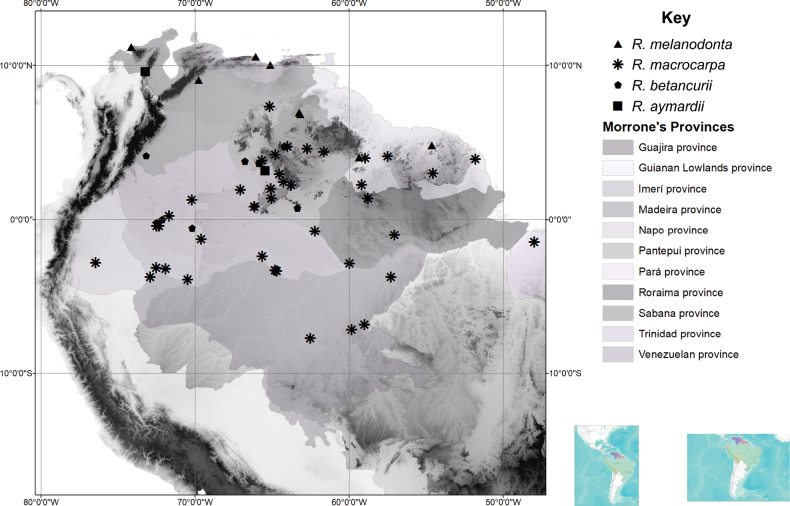
Distribution of *R.melanodonta* (triangles), *R.macrocarpa* (asterisks), *R.betancurii* (pentagons), *Rinoreaaymardii* (squares).

*Rinoreaaymardii*, which is only known from the type specimen, occurs the Alto Orinoco–Casiquiare Biosphere Reserve in Amazonas State, Venezuela, a biogeographic area classified as the Province of Imerí in the Boreal Brazilian Dominion (*sensu*Morrone (2014)). It grows in terra firma humid forest at elevations of 100–180 m. The landscape of the type locality is characterised by gently sloping (10–20%) hills composed of granitic and quarzitic rocks (Aymard 2000). (Fig. 4)

#### Etymology.

*Rinoreaaymardii* honors the Venezuelan botanist Gerardo Aymard, who has made significant contributions to the taxonomic knowledge of the flora of Venezuela, especially the south-western part of the country in the Río Negro and Orinoco River Basins.

#### Phenology.

The species is only known from one flowering specimen collected in February.

#### Conservation status.

*Rinoreaaymardii* has a geographic range in the form of an EOO of 4 km^2^ and AOO of 4 km^2^. It is currently known only from the type collection, which represents one location within the terra firma forests of the Alto Orinoco–Casiquiare Biosphere Reserve in Venezuela where it is threatened by illegal logging and mining. Based on its limited AOO, the single location and the projected continuing decline in the quality of habitat due to uncontrolled deforestation and resource extraction, the species might be preliminarily assigned to the “Critically Endangered.” However, given the difficulty accessing this inadequately explored area, it is likely there are additional occurrences that would allow a more accurate assessment of the risk of extinction than is possible at this time. The species is therefore preliminarily assigned to the IUCN (2012) Category of “Data Deficient” [DD].

#### Notes.

*Rinoreaaymardii* is sympatric with *R.macrocarpa* from which it can be differentiated by the smaller lamina 4–11 × 1–2.7 cm with crenate margins (vs. larger lamina 5.5–21.5 × 2–9.2 cm with subentire to subcrenate margins in *R.macrocarpa*), filaments free (vs. filament fused into a tube) and style filiform (vs. style conical).

### ﻿3. Rinorea chiribiquetensis

Taxon classificationPlantaeMalpighialesViolaceae

Hoyos-Gómez sp. nov.

E069FC9C-F725-5A7E-8EA0-0CC3712C3E64

urn:lsid:ipni.org:names:77342873-1

#### Type.

Colombia. Dept. Caquetá: alrededores del refugio la Selva, 1°4'0.23"N, 72°44'24.6"W, 630 m elev., 1 Dec 1992, *M. Velayos et al. 6509* (holotype: MO [acc. 4644279; barcode MO-1590549]!; isotypes: COL!, MA [cat. 543845]!).

#### Description.

*Rinoreachiribiquetensis* is similar to *R.ovalifolia* (Britton) S.F.Blake by the lamina base symmetrical, margin subserrate, ovary globose and style filiform, but it differs by the smaller lamina, 3.2–7.2 × 1.6–3.6 cm, with appressed pubescence on the abaxial surface and domatia present (vs. the larger lamina, 3–16 × 2–7.5 cm, glabrescent and lacking domatia in *R.ovalifolia*), shorter inflorescence, 1.5–5 cm long (vs. longer inflorescence, 4–12 cm long), shorter pedicels, 1.7–3.3 mm long (vs. longer pedicels, 4–6 mm long) and stamens with filaments free (vs. stamens with filaments fused).

**Figure 5.**
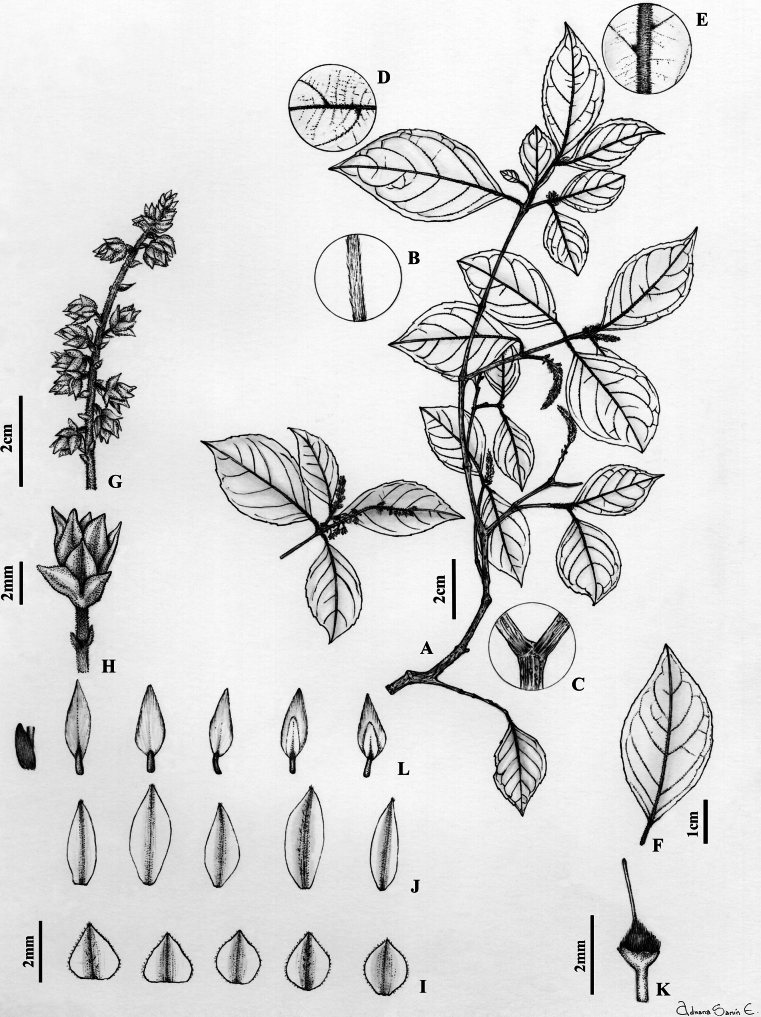
*Rinoreachiribiquetensis* Hoyos-Gómez **A** habit **B** detail of young twig with pubescence **C** detail of young twig showing lenticels **D** detail of leaf showing pubescence, adaxial surface **E** detail of leaf showing domatia, abaxial surface **F** leaf architecture **G** infructescence **H** flower **I** sepals, abaxial surface **J** petals, abaxial surface **K** gynoecium **L** stamens, showing adaxial and abaxial surfaces, with a detail of the dorsal gland (left). (**A–L**: *M. Velayos 6509* [MO)].

Shrubs to 1.5 m tall, terminal branchlets pubescent with appressed golden trichomes 0.3 mm long, lenticels 0.5–0.6 mm. Leaves opposite, petiolate; petiole 3–6 mm long, pubescent with erect golden trichomes 0.2–0.3 mm long; stipules deciduous, free, lanceolate 1.9–2.1 × 0.9–1 mm, pubescent with appressed golden trichomes 0.2 mm long, margin entire; lamina elliptic, 3.2–7.2 × 1.6–3.6 cm, adaxially pubescent on mid-vein with appressed golden trichomes 0.2–0.3 mm long, abaxially pubescence on mid-vein, secondary and tertiary veins with appressed golden trichomes 0.2–0.3 mm long; with 5–6 major secondary vein pairs, semi-craspedodromous; secondary veins with irregular spacing, and veins angle smoothly decreasing proximally between them; symmetrical base, cuneate, margin subserrate, apex acuminate, acumen 5–6 mm long, mucronate, leaf domatia present. Inflorescence axillary, lateral or terminal, racemose, 1.5–5 cm long, 0.5–1 cm diam., axis pubescent with erect golden trichomes 0.2 mm long; pedicels 1.7–3.3 mm long, articulated near the middle, pubescent with erect golden trichomes 0.2 mm long,; bractlets persistent below articulation, subopposite, ca. 1 × 1 mm, herbaceous, costa pubescent with appressed golden trichomes 0.2 mm long, 3–5 veins; peduncle bracts deciduous, narrowly triangular 1–1.4 × 1–1.5 mm, herbaceous, pubescent with appressed golden trichomes 0.2 mm long, 3–5 veins, margin ciliolate. Flowers 3.2–3.5 × 3–3.3 mm; sepals subequal in size and shape, triangular, 1.5–1.7 × 1.7–1.9 mm, 5–7 veined, costa pubescent with appressed golden trichomes 0.2 mm long, margin ciliolate, apex apiculate; petals lanceolate, 3–3.3 × 1.4–1.6 mm, pubescent with erect golden trichomes 0.2 mm long near the apex, margin entire, apex acute; stamens 2.6–3 mm long, filaments free, 0.7–0.9 mm, dorsal gland glabrous, completely covering the filament; anthers elliptic, 1–1.1 × 0.3–0.5 mm, glabrous, apex rounded, connective 0.6–0.7 mm long, dorsal connective scale lanceolate, 2.2–2.4 × 0.8–1 mm, scarious, margin subentire, brown-orange; ovary globose, 0.7–0.8 × 0.8–0.9 mm, pubescent with appressed golden trichomes 0.4 mm long; style erect, filiform, 1.7–2 × ca. 0.1 mm, glabrous, stigma acute. Fruit unknown. (Fig. 5)

#### Distribution and habitat.

**Figure 6.**
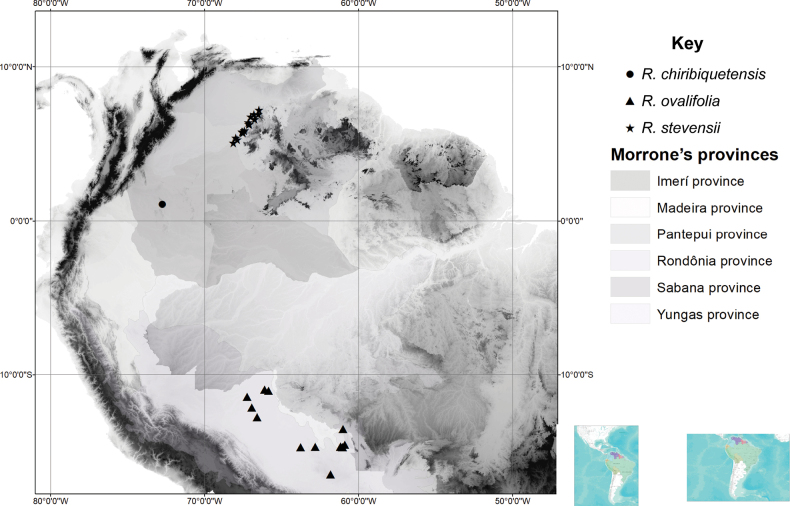
Distribution of *Rinoreachiribiquetensis* (circle), *R.ovalifolia* (triangles), *R.stevensii* (stars).

*Rinoreachiribiquetensis* is endemic to Caquetá Department, Colombia, an area belonging to the Biogeographical Province of Imerí in the Boreal Brazilian Dominion (sensu Morrone (2014)). It occurs in lowland tropical rainforest from rocky substrates at an elevation of 630 m. (Fig. 6)

#### Etymology.

*Rinoreachiribiquetensis* is named for the Chiribiquete National Park in Colombia.

#### Phenology.

The species is only known from one flowering specimen collected in December.

#### Conservation status.

The region encompassing the vast and remote Chiribiquete National Park is relatively little explored as evidenced by the scarcity of collections in the Tropicos database (https://www.tropicos.org; accessed 25 February 2023), GBIF (https://www.gbif.org; accessed 25 February 2023) and other Colombian Herbaria (e.g. COAH, COL, HUA). There are insufficient data to assess the risk of extinction of *Rinoreachiribiquetensis* and, therefore, the species is preliminarily assigned to the IUCN (2012) category of “Data Deficient” [DD].

#### Notes.

*Rinoreachiribiquetensis* is sympatric with *R.macrocarpa*, but it can be differentiated by the presence of domatia (vs. domatia absent in *R.macrocarpa*), smaller lamina, 3.2–4.2 × 1.6–3.6 cm (vs. larger lamina, 5.5–21.5 × 2–9.2 cm), filaments free (vs. filaments fused into a tube) and style filiform (vs. style conical). *Rinoreachiribiquetensis* can be differentiated from *R.stevensii* by the smaller lamina, 3.2–4.2 × 1.6–3.6 cm (vs. larger lamina, 4–12.5 × 2.5–7 cm in *R.stevensii*) and less dense pubescence on the abaxial lamina surface, with trichomes 0.2–0.3 mm long (vs. denser pubescence on the lamina, with trichomes 0.4–0.5 mm long).

### ﻿4. Rinorea stevensii

Taxon classificationPlantaeMalpighialesViolaceae

Hoyos-Gómez sp. nov.

7148687E-7473-5FCF-9B04-DF64A0C73B6E

urn:lsid:ipni.org:names:77342874-1

#### Type.

Colombia. Dept. Vichada: Parque Nacional “El Tuparro,” piedra canal near the south end of airstrip at Centro Administrativo, 5°17'N, 67°52'W, ca. 100 m elev., 5 Mar 1985, *J. L. Zarucchi et al. 3577* (holotype: NY [barcode 04205808]!; isotypes: COAH [cat. 24597]!, COAH [cat. 55474]!, FMB [cat. 4307]!, FMB [cat. 21506]!, MO [acc. 3499269; barcode MO-1592746]!, U [barcode U1766360]!).

#### Description.

*Rinoreastevensii* is similar to *R.ovalifolia* by the lamina base symmetrical, style erect and two glabrous seeds per valve, but it differs by the lamina abaxial surface with golden pubescence (vs. the lamina abaxial surface glabrescent in *R.ovalifolia*), domatia present (vs. domatia absent), inflorescences cylindrical, 2–5 cm long (vs. inflorescence conical, 4–12 cm long), costa of petals and sepals pubescent (vs. costa of petals and sepals glabrescent), filaments free (vs. filaments fused), and capsule pubescent (vs. capsule glabrescent).

**Figure 7.**
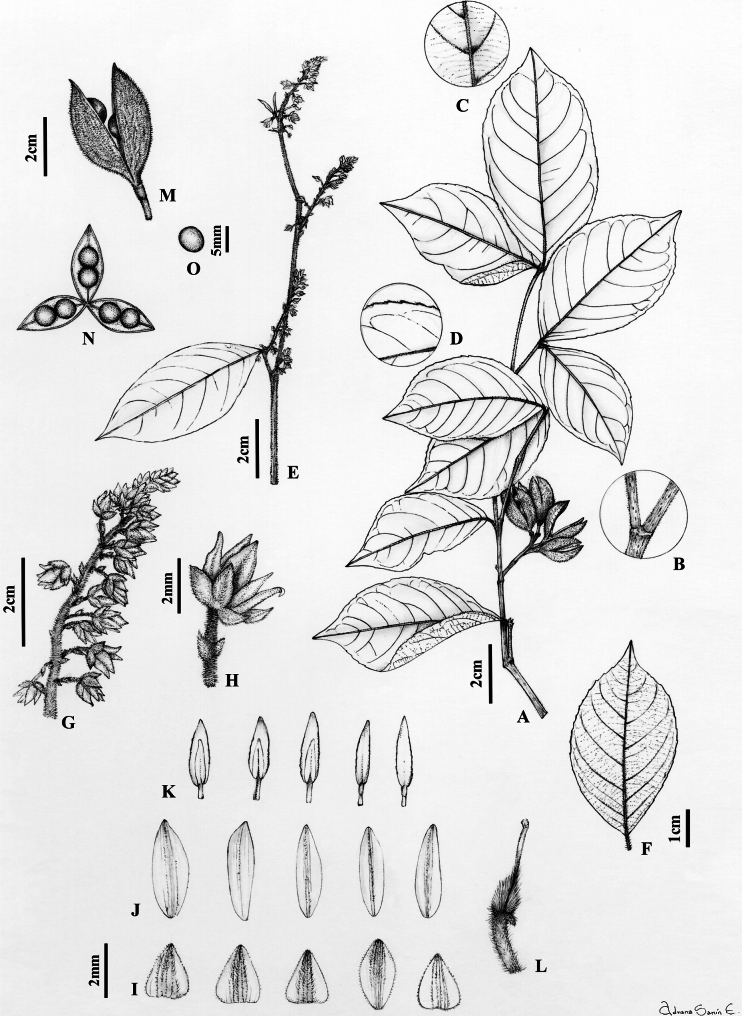
*Rinoreastevensii* Hoyos-Gómez **A** habit **B** detail of young twig showing lenticels and pubescence **C** detail of leaf showing domatia, abaxial surface **D** detail of leaf showing margin ciliate, adaxial surface **E** branchlet with inflorescences **F** leaf architecture **G** infructescence **H** flower **I** sepals, abaxial surface **J** petals, abaxial surface **K** stamens, showing adaxial and abaxial surfaces **L** gynoecium **M** fruit **N** detail of fruit showing two seeds per valve **O** seed. (**A–D**: *B. M. Boom 6564* [MO]; **E–O**: *J. L. Zarucchi 3577* [MO]).

Trees 1.5–4 m tall, terminal branchlets pubescent with erect golden trichomes 0.4–0.5 mm long. Leaves opposite, petiolate; petiole 2–6 mm, pubescent with long golden trichomes 0.3–0.4 mm long; stipules deciduous, free, lanceolate, herbaceous, 2.5–5 × 1–1.5 mm, pubescent with appressed golden trichomes 0.4–0.5 mm long; margin entire; lamina elliptic, 4–12.5 × 2.5–7 cm, adaxially pubescent on mid-vein and abaxially pubescent on mid-vein and secondary veins with erect golden trichomes 0.4–0.5 mm long; semi-craspedodromous, with 6–9 major secondary vein pairs, secondary veins with irregular spacing and vein angles smoothly decreasing proximally between them, base rounded, symmetrical, margin crenate, ciliate, apex acuminate, acumen 0.5–1 cm long, mucronate, domatia present. Inflorescence axillary, lateral or terminal, racemose, 2–5 cm long, 1–1.5 cm diam., axis pubescent with erect golden trichomes 0.4–0.5 mm long; pedicels 2.5–6.5 mm, pubescent with erect golden trichomes 0.4–0.5 mm long, articulated near the middle; bractlets persistent below articulation, subopposite, ca. 1 × 1 mm, herbaceous, costa pubescent with appressed golden trichomes 0.4–0.5 mm long; peduncle bracts persisting, broadly triangular, 1–1.5 × 1–1.5 mm, herbaceous, pubescent with appressed golden trichomes 0.4–0.5 mm long, margin ciliolate. Flowers 2.8–3.8 × 3–3.4 mm long, sepals subequal, triangular, 2–2.5 × 1–1.6 mm, 7–11-veined, pubescent with appressed golden trichomes 0.5 mm long, margin ciliolate; petals lanceolate, 2.8–4.3 × 1.2–1.9 mm, costa pubescent with appressed golden trichomes 0.3–0.4 mm long, margin entire, cream to yellow in vivo, pale brown when dry; stamens 2.5–3 mm long, all filaments free, 0.4–0.6 mm, dorsal gland covering completely the filament, glabrous; anthers elliptic, 1.4–1.7 × 0.3–0.8 mm, apex obtuse, connective 0.8–0.9 mm, dorsal connective scale lanceolate, 2.4–2.5 × 0.9–1.0 mm long, margin subentire, scarious, orange-brown; ovary globose, 0.8–1.5 × 0.7–1.3 mm, pubescent with appressed golden trichomes 0.5 mm long; style erect, filiform, 1.8–2.4 × ca. 0.1 mm long, pubescent proximally with appressed trichomes 0.5 mm long, stigma acute. Fruit a symmetrical, subligneous capsule dehiscent along three sutures, ellipsoid, 1.5–2.3 × 0.5–0.6 cm, acuminate at the apex, veined, pubescent with erect golden trichomes 0.5 mm long, green when fresh, brown when dry. Seeds two per valve, globose, 4–5 mm diam., glabrous, without maculae, brown when dry. (Fig. 7)

#### Distribution and habitat.

*Rinoreastevensii* occurs in Colombia in the border region with Venezuela near the Orinoco River and in El Tuparro National Park. It also occurs in Venezuela in the vicinity of Puerto Ayacucho. This distribution corresponds to the Biogeographical Provinces of Pantepui and Sabana, in the Boreal Brazilian and Pacific Dominions, respectively (*sensu*Morrone (2014)). The species grows on granitic hills and slopes in lowland semi-deciduous tropical rainforests and savannahs, at elevations of 85–520 m. (Fig. 6)

#### Etymology.

*Rinoreastevensii* is named in honour of Dr. Peter Stevens, Professor of Biology at the University of Missouri Saint-Louis and curator of the Missouri Botanical Garden. Prof. Stevens was the major advisor for SEHG’s master’s degree.

#### Vernacular names.

*Aruni yó* (*B. M. Boom et al. 6564*).

#### Conservation status.

*Rinoreastevensii* has a geographic range in the form of an EOO of 5,070 km^2^ and AOO of 56 km^2^. It is represented by two locations within the El Tuparro National Park in Colombia and eight locations outside of protected areas in Venezuela where it is threatened by deforestation, illegal resource extraction and uncontrolled fires. Based on its limited AOO, the number of locations and the projected continuing decline in the quality of habitat, the species is preliminarily assigned to the “Vulnerable” category [VU B2ab(iii)].

#### Notes.

Based on several morphological differences and an allopatric distribution, *Rinoreastevensii* is segregated from *R.ovalifolia* (as circumscribed by Hekking (1988)). Hekking noted the variable morphology of some specimens now representing *R.stevensii*, but he was unsure of their placement: “The leaves of *Rinoreaovalifolia* are variable in character, e.g. the underside of the leaves varies from densely hispidulous to glabrescent and domatia may be present or not”. Hekking invoked introgressive hybridisation between *R.ovalifolia* and R.pubifloravar.pubiflora to explain the morphological variability, but we disagree with his opinion. We hypothesise that the distinctive morphological characteristics and the discrete biogeographic distribution suggest that *R.stevensii* is a separately evolving lineage (*sensu* De Queiroz (2007)) making it worthy of recognition at the rank of species. Key morphological differences amongst *R.stevensii*, *R.ovalifolia* and R.pubifloravar.pubiflora are presented in Table 1.

**Table 1.** Key morphological differences among *R.stevensii*, *R.ovalifolia*, and *R.pubiflora*.

Character	*R.stevensii*	*R.ovalifolia*	R.pubifloravar.pubiflora
Lamina base	Rounded	Decurrent	Decurrent
Lamina pubescence	Pubescent	Glabrescent	Glabrous on both surfaces
Domatia	Present	Absent	Present
Inflorescence length	2–5 cm	4–12 cm	3–7 cm
Petal pubescence	Glabrous with costa pubescent	Glabrescent	Glabrous with costa pubescent
Setal pubescence	Glabrous with costa pubescent	Glabrescent	Densely pubescent
Filament fusion	Not fused	Fused to form a staminal tube	Not fused
Seeds per valve	2	2	3

In addition to an allopatric distribution, *Rinoreastevensii* can be differentiated from *R.chiribiquetensis* (the other species segregated from *R.ovalifolia*) by several morphological characters. It has longer and more abundant pubescence on the lamina, with trichomes 0.4–0.5 mm long (vs. shorter and less abundant pubescence on the lamina, with trichomes 0.2–0.3 mm long in *R.chiribiquetensis*), larger lamina, 4–12.5 × 2.5–7 cm (vs. smaller lamina, 3.2–4.2 × 1.6–3.6 cm) and larger petals 2.8–4.3 × 1.2–1.9 mm (vs. 3–3.3 × 1.4–1.6 mm). Rinoreapubifloravar.pubiflora co-occurs with *R.stevensii*, but it is differentiated by having three pubescent seeds per valve, whereas *R.stevensii* has two glabrous seeds per valve.

#### Additional specimens examined.

**Colombia. Dept. Vichada**. Mpio. de Puerto Carreño: Cerro al N del Centro Adm. Inderena, 5°21'17.0"N, 67°51'40.6"W, 4 Apr 1995 (fr), *M. P. Córdoba et al. 1266* (COAH, COL, FMB); base del cerro Rocoso, 100 m elev., 8 Oct 1979 (fl), *P. Vincelli 1047* (COAH, COL, FMB). **Venezuela. Estado Amazonas.** Mpio. De Puerto Ayacucho: 35 km. south of Puerto Ayacucho, at the “Tobagón”. Large igneous outcrop bordering forest on slope, 85 m elev., 4 May 1977 (fr), *J. Steyermark & O. Huber 113844* (COL, MO). Mpio. de Atures: 23 km NE of Puerto Ayacucho and 10 km E of the highway, hills and base of hills, near Cachama, 90 m elev., 5°51'N, 67°24'W, 90 m elev., 19 Apr 1978 (fr), *G. Davidse et al. 15300* (MO, NY, U, US); 14–15 km NE of Puerto Ayacucho, along road to “El Burro,” 5°47'N, 67°32'W, 85 m elev., 15 Apr 1978 (fr), *G. Davidse et al. 15070* (MO, NY, US); alrededores de Puerto Ayacucho +/- 11 km N, 5°44'N, 67°30'W, 15 Apr 1978 (fl), *O. Huber et al. 1450* (U, US); *ibid*., 5°43'N, 67°30'W, 29 Jan 1978 (fl), *O. Huber et al. 1502* (K, NY, U, US); Piedra el Berraco, laja granítica que conduce a Provincial, 10 km al NE de Puerto Ayacucho, 5°47'N, 67°34'W, Apr 1997 (fl, fr), *Á. Fernández-Pérez et al. 10800* (US); E of río Parguaza, 125 km N of Puerto Ayacucho, 6°17'N, 67°5'W, 11 Sep 1985 (st), *J. A. Steyermark et al. 131751* (MO, U); Parguaza, 22 Apr 1946 (fr), *I. Velez 2448* (US). **Estado Bolivar.** Mpio. Cedeño: vicinity of Panare village of Corozal, 6 km from Manipure towards Caicara, 6°55'N, 66°30'W, 24 Sep 1985 (st), *B. M. Boom et al. 6083* (NY); *ibid*., 6°55'N, 66°30'W, 90 m elev., 19 Apr 1986 (fr), *B. M. Boom & M. Grillo 6564* (MO, F, U, US); carretera Caicara–El Burro, 16 Apr 1984 (fl), *B. Stergios et al. 8494* (MO); Caicara 100 m elev., 10 Jun 1940 (st), *L. W. Williams 13255* (F). Mpio. Foráneo La Urbana: cerca a la desembocadura del río Orinoco, 6°46'N, 67°00'W, 31 Jan 1989 (fl), *N. Cuello 718* (MO, U, US); Los Pijiguaos, afloramiento granítico 1.5 km al N del campamento Bauxiven, 6°35'N, 66°47'W, 7 Aug 1993 (st), *A. Gröger et al. 1077-B* (MO); E slopes of cerro Pijiguao (N end of serrania Suapure) above Pijiguao, ca. 70 km from mouth of Río Suapure, 110–520 m eelv., 19 Jan 1956 (fl), *J. J. Wurdack et al. 41295* (F, K, MG, MO, NY, U, US).

### ﻿5. Rinorea cogolloi

Taxon classificationPlantaeMalpighialesViolaceae

Hoyos-Gómez sp. nov.

8F21BF95-FF51-580D-ABFC-81FB36580053

urn:lsid:ipni.org:names:77342875-1

#### Type.

Colombia. Dept. Antioquia: Mpio. de San Luis, Cañón del Río Claro, sector norte, margen izquierda, 5°53'N, 74°39'W, 340–500 m elev., 24 Dec 1983, *A. Cogollo 1075* (holotype: HUA [032011]!; isotypes: COL [299700]!, MO [acc. 3737288; barcode MO-1590440]!).

#### Description.

*Rinoreacogolloi* is similar to *R.hirsuta* by the elliptic lamina with ferruginous trichomes and lacking domatia, but it differs by the asymmetrical lamina base (vs. lamina base symmetrical in *R.hirsuta*), inflorescence thyrsoid (vs. inflorescence racemose), petals 2–3 × 0.7–1.3 mm (vs. 4–4.3 × 1.5–1.8 mm), stamens 1.7–2 mm long (vs. 3–3.3 mm long), dorsal gland pubescent (vs. dorsal gland glabrous), seeds pubescent (vs. glabrous) and capsule asymmetrical and pilose (vs. capsule symmetrical and velutinous).

**Figure 8.**
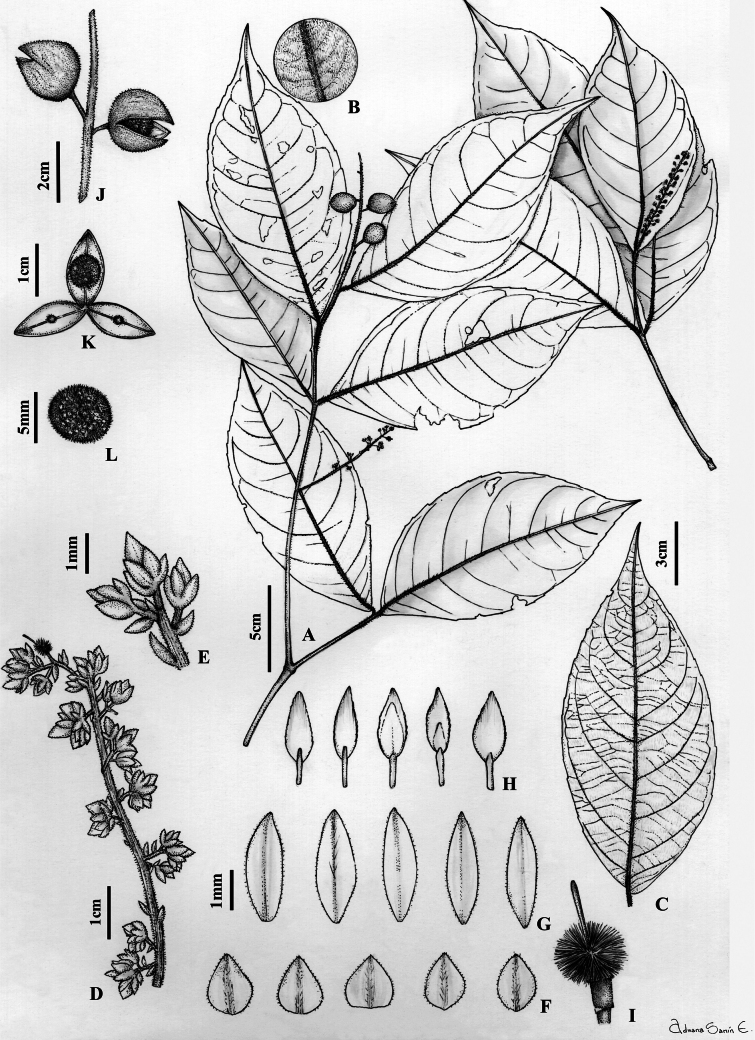
*Rinoreacogolloi* Hoyos-Gómez **A** habit **B** detail of leaf pubescent, abaxial surface **C** leaf architecture **D** infructescence **E** cymule with flowers **F** sepals, abaxial surface **G** petals, abaxial surface **H** stamens, showing adaxial and abaxial surfaces **I** gynoecium **J** infructescence **K** detail of fruit showing one seed per valve **L** seed. (**A–L**: *A. Cogollo 786* [MO]).

Shrubs or trees 2–6 m tall, terminal branchlets pubescent with erect ferruginous trichomes 0.4–0.6 mm long. Leaves opposite, petiolate; petiole 1.9–5 mm, pubescent with erect ferruginous trichomes 0.3–0.4 mm long; stipules deciduous, free, lanceolate, membranous, 1.9–2.5 × 0.7–1 mm, pubescent with appressed ferruginous trichomes 0.2–0.3 mm long, margin ciliolate; lamina elliptic, 7–17 × 2.5–7 cm long, herbaceous, adaxially pubescent on mid-vein with erect ferruginous trichomes 0.2–0.3 mm long, abaxially pubescent with erect ferruginous trichomes 0.4–0.5 mm long, with 5–8 major secondary vein pairs, semi-craspedodromous, secondary veins with regular spacing and angled to the mid-vein, base rounded, asymmetrical, margin crenate, apex acuminate, acumen 0.5–1 cm long, mucronate, leaf domatia absent. Inflorescence axillary, lateral or terminal, thrysoid, 4–8 cm long, 0.5–1 cm diam., axis pubescent with erect ferruginous trichomes 0.2–0.3 mm long; cymules 3–5 flowered; common peduncule 1–1.5 mm, pubescent with erect ferruginous trichomes 0.2 mm long; pedicels 0.5–1 mm, articulated at the base, pubescent with erect ferruginous trichomes 0.2 mm long; bractlets alternate, persistent below articulation, ca. 0.5 × 0.5 mm, herbaceous, costa pubescent with appressed ferruginous trichomes 0.2 mm long, margin ciliolate; peduncle bracts persistent, broadly triangular, 1–1.5 × 1–1.5 mm, herbaceous, costa pubescent with appressed ferruginous trichomes 0.3–0.4 mm long; margin ciliolate. Flowers 2.5–2.8 × 2.5–2.8 mm, sepals subequal in size and shape, triangular, 1–1.5 × 0.5–0.7 mm, pubescent with appressed ferruginous trichomes 0.2 mm long, margin ciliolate; petals elliptic, 2–3 × 0.7–1.3 mm long, pubescent with appressed ferruginous trichomes 0.2 mm long, margin ciliolate, cream to yellow in vivo, brown when dry; stamens 1.7–2 mm long, filaments free, glabrous, 0.9–1 mm, dorsal gland covering the filament, pubescent with spreading ferruginous trichomes 0.2 mm long; anthers elliptic, 0.6–1 × 0.4–0.6 mm, pubescent with spreading ferruginous trichomes 0.2 mm long, apex obtuse, connective 0.6–0.7 mm, pubescent with appressed ferruginous trichomes 0.2 mm long, dorsal connective scale lanceolate, 1.2–1.6 × 0.7–0.9 mm, scarious, margin subentire, orange-brown; ovary globose, 1–1.5 mm diam., pubescent with erect ferruginous trichomes 0.3–0.5 mm long; style erect subclavate, filiform, 1.5–1.8 × 0.1 mm, stigma acute. Fruit an asymmetrical, subligneous capsule dehiscent along three sutures, ellipsoid, 0.8–1.5 × 0.5–0.6 cm, apex acuminate, veined, pubescent with erect ferruginous trichomes 0.2–0.3 mm long, green when fresh, brown when dry. Seeds one per valve, globose, 5–7 mm diam., with maculae, pubescent with erect ferruginous trichomes 0.3 mm long, brown when dry. (Fig. 8)

#### Distribution and habitat.

**Figure 9.**
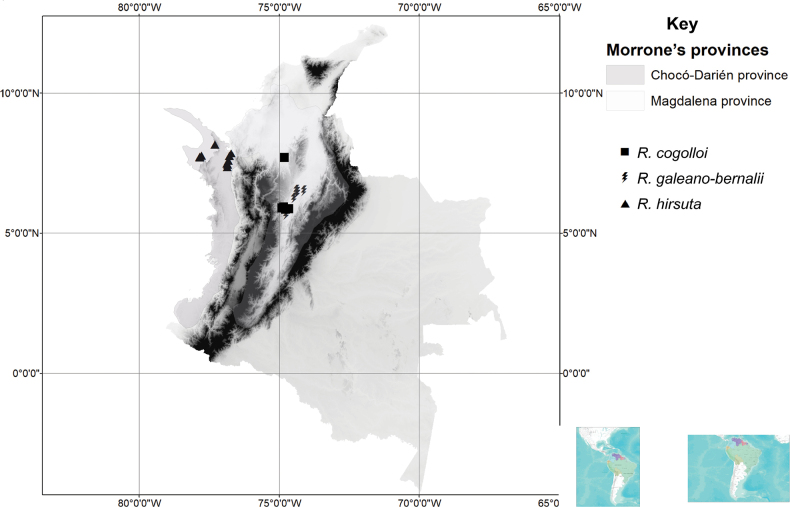
Distribution of *Rinoreacogolloi* (squares), *R.galeanoae-bernalii* (bolts), *R.hirsuta* (triangles).

*Rinoreacogolloi* is narrowly distributed in Antioquia Department, Colombia, an area that coincides with the Biogeographical Province of Magdalena in the Pacific Dominion (sensu Morrone 2014). The species grows in lowland tropical rainforests in a region characterised by a karstic topography, occurring at elevations of 30–500 m. (Fig. 9)

#### Etymology.

*Rinoreacogolloi* is named in honour of Professor Alvaro Cogollo who has made contributions to the knowledge of Magnoliaceae taxonomy and the flora of Colombia, especially the middle Magdalena River Valley, where the species is endemic.

#### Phenology.

The species flowers between August and December; fruiting specimens were collected in February and August through October.

#### Conservation status.

*Rinoreacogolloi* has a geographic range in the form of an EOO of 1,641 km^2^ and AOO of 32 km^2^. It is currently known from eight locations outside of protected areas. The region, underlain by karstic rocks, is heavily impacted by limestone extraction for concrete production and uncontrolled deforestation for grazing, agriculture and logging. Another location in north-eastern Antioquia Department is threatened with gold mining and deforestation. Given the high demand for cement products, the generally poor management of forest resources in the area and the number of locations, we project a continuing decline in the extent and quality of habitat for the species. *Rinoreacogolloi* is preliminarily assigned to the “Vulnerable” category [VU B1+B2ab(iii)] in accordance with the IUCN Red List Categories and Criteria (IUCN 2012).

#### Notes.

Hekking (1988) noted the aberrant lamina pubescence of several specimens of *Rinorealindeniana* (e.g. *A. Cogollo 786*, *1075*, *1483*). *Rinoreacogolloi* and *R.lindeniana* share several characteristics, such as leaf venation architecture and asymmetrical lamina bases, but *R.cogolloi* is readily separated from *R.lindeniana* by the densely pubescent indument on the abaxial lamina surface (vs. the abaxial lamina surface pilose to glabrescent in *R.lindeniana*). *Rinoreacogolloi* occurs sympatrically with *R.galeanoae-bernalii* (see below), but *R.cogolloi* has densely pubescent indument on the abaxial lamina surface (vs. abaxial lamina surface glabrescent in *R.galeanoae-bernalii*), pubescent fruit with spreading, erect ferruginous trichomes 0.3–0.4 mm long (vs. densely tomentose fruit) and the seeds pubescent with erect ferruginous trichomes 0.3 mm long (vs. seeds glabrous). *Rinoreacogolloi* and *R.hirsuta* have been confused by the similarity of the leaves and indumenta, but they can be differentiated by the asymmetrical lamina base, thyrsoid inflorescence, dorsal gland pubescent, pubescent seeds and capsule asymmetrical and pilose. In addition, these two species have different distributions, with *R.cogolloi* occurring in the Magdalena Province and *R.hirsuta* restricted to the Chocó-Darien Province (sensu Morrone (2014)). *Rinoreacogolloi* is also similar to R.pubifloravar.pubiflora, but it can be differentiated by its valves containing one seed (vs. valves containing three seeds in *R.pubiflora*) and the thyrsoid inflorescence (vs. racemose inflorescence). Table 2 highlights the key morphological differences amongst these five species.

**Table 2.** Key morphological differences among *R.cogolloi*, *R.lindeniana*, *R.galeanoae-bernalii*, *R.hirsuta*, and *R.pubiflora*.

Character	*R.cogolloi*	*R.lindeniana*	*R.galeanoae-bernalii*	*R.hirsuta*	R.pubifloravar.pubiflora
Lamina base	Rounded	Rounded	Decurrent		Decurrent
Lamina pubescence	Adaxial surface glabrous, pubescent midvein; abaxial surface densely pubescent	Both surfaces glabrescent	Adaxial surface glabrous, pubescent midvein; abaxial surface pubescent to glabrescent	Adaxial surface glabrous, pubescent midvein; abaxial surface densely pubescent	Glabrous on both surfaces
Inflorescence	Thrysoid	Pseudo-racemose (basal portions of inflorescence with lateral cymules)	Racemose	Racemose	Racemose
Inflorescence length	4–8 cm	5–15 cm	3–10 cm	5–10 cm	3–7 cm
Petal pubescence	Pubescent	Pubescent	Glabrous with costa pubescent	Glabrous with apex pubescent	Glabrous with costa pubescent
Setal pubescence	Pubescent	Pubescent	Glabrous with costa pubescent distally	Glabrous with costa and apex pubescent	Pubescent
Filament fusion	Not fused	Not fused	Fused to form a staminal tube	Not fused	Not fused
Seeds per valve	1	1	1	1	3
Seed pubescence	Pubescent	Pubescent	Glabrous	Glabrous	Pubescent

#### Additional specimens examined.

**Colombia. Dept. Antioquia.** Mpio. de Caucasia: Vda. la Arenosa, km 45 vía al Bagre, Finca la Natalia, 7°47'46"N, 74°53'9"W, 70–100 m elev., 26 Aug 2022 (st), *S. E. Hoyos-Gómez et al. 5184* (HUA, UBDC); *ibid.*, 7°47'59"N, 74°52'45"W, 80 m elev., 10 Nov 2022 (fr), *M. Montoya M9004* (HUA); Mpio. de Nechí: Vda. El Catorce, entre la mina el 14 y la roca del camino, 8°94'60"N, 74°46'14"W, 30–40 m elev., 6 Mar 2010 (fl), *W. Rodríguez 6599* (COL, MEDEL); Mpio. de Puerto Triunfo: alrededores de la Gruta “El Condor,” 5°56'N, 74°50'W, 12 Oct 1983 (fl, fr), *A. Cogollo 786* (COL, HUA, MO); *ibid.*, 22 Oct 1989 (fr), *J. G. Ramírez 4263* (JAUM); Río Claro carretera al Cairo, 17 Sep 1982 (fl, fr), *E. Rentería 2722* (JAUM, MO). Mpio. de Sonsón: vereda Jerusalén, 5°55'00"N, 74°51'00"W, 200 m elev., 24 Dec 2010 (st), *S. E. Hoyos-Gómez et al. 1145* (HUA, JAUM, MO); *ibid*., 5°55'00"N, 74°51'00"W, 200 m elev., 24 Dec 2010 (fl), *S. E. Hoyos-Gómez et al. 1152* (HUA, JAUM); *ibid*., 5°55'00"N, 74°51'00"W, 200 m elev., 24 Dec 2019 (fl), *S. E. Hoyos-Gómez & G. A. Wahlert 3780* (HUA, JAUM); *ibid*., 5°53'30"N, 74°50'58"W, 200 m elev., 23 Sep 2018 (fl, fr), *J. P. Tobón et al. 2798* (HUA, JAUM); vereda Jerusalén, 5°53'30"N, 74°50'58"W, 380 m elev., 23 Sep 2018 (fr), *J. P. Tobón et al. 2799* (HUA, JAUM); *ibid*., 5°53'39"N, 74°51'08"W, 380 m elev., 24 Sep 2018 (fl, fr), *J. P. Tobón et al. 2803* (HUA, JAUM); *ibid*., 5°53'25"N, 74°50'50"W, 24 Sep 2018 (fl, fr), *J. P. Tobón et al. 2806* (HUA, JAUM); *ibid*., 5°53'44.1"N, 74°51'18"W, 1 Feb 2019 (fr), *J. P. Tobón et al. 2897* (JAUM); vereda Jerusalén, Reserva Natural Cañón del Río Claro, “El Refugio”, margen derecha, 5°53.759'N, 74°51.219'W, Apr 2013 (fr), *L. Cano et al. 43* (HUA); corregimiento Jerusalén, vía Medellín-Bogotá, predios de Sumicol, cuenca del Río Claro, en cercanías de la torre de energía T 66, 5.915756°N, 74.849186°W, 4 Aug 2020 (fl, fr). *J. M. Velez et al. 7388* (MEDEL, COL); *ibid*., 5.916104°N, 74.849473°W, 17 Sep 2020 (fl), *J. M. Velez et al. 7511* (JAUM); *ibid*., 5.916182°N, 74.850481°W, 16 Feb 2021 (fr). *J. M. Velez et al. 7604* (JAUM).

### ﻿6. Rinorea galeanoae-bernalii

Taxon classificationPlantaeMalpighialesViolaceae

Hoyos-Gómez sp. nov.

4FDAC500-2BB3-59F2-B895-778E120E0CEE

urn:lsid:ipni.org:names:77342876-1

#### Type.

Colombia. Dept. Caldas: La Dorada, vereda la Atarraya, finca los Achiles, relictos de bosque al margen trasera de la finca, 5°40'20.3"N, 74°44'26.4"W, 214 m elev., 3 Aug 2021 (fl), *D. Sanín et al. 7966* (holotype: HUA [acc. 225716]!; isotype: FAUC!).

#### Description.

*Rinoreagaleanoae-bernalii* is similar to *R.hirsuta* by the elliptic lamina with symmetrical base and lacking domatia, inflorescence racemose, capsule symmetrical and pubescent with velutinous trichomes and one glabrous seed per valve, but it differs by the abaxial surface of the lamina glabrescent (vs. abaxial surface of the lamina pubescent with ferruginous trichomes in *R.hirsuta*), filaments fused at the base forming a staminal tube (vs. filaments free not forming a staminal tube), anthers with pubescence between the thecae (vs. anthers glabrous between the thecae) and style proximally pubescent (vs. style proximally glabrous).

**Figure 10.**
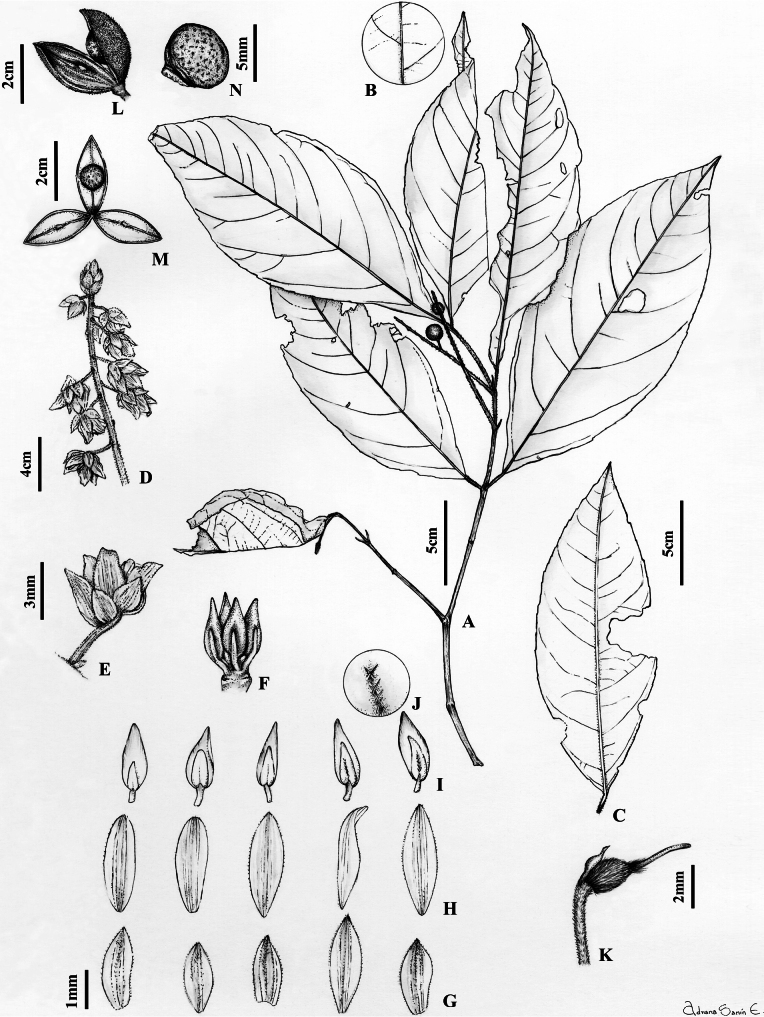
*Rinoreagaleanoae-bernalii* Hoyos-Gómez **A** habit **B** detail of leaf, abaxial surface **C** leaf architecture **D** infructescence **E** flower **F** detail of filaments fused at the base forming a staminal tube **G** sepals, abaxial surface **H** petals, abaxial surface **I** stamens, adaxial and abaxial surfaces **J** detail of anthers showing pubescence between the thecae **K** gynoecium **L** fruit **M** detail of fruit showing one seed per valve **N** seed. (**A–E, N**: *D. Sanin 7704* [HUA]; *D. Sanin 7966* [HUA]).

Treelets 1.5–4 m tall, terminal branchlets pubescent with erect golden trichomes 0.2–0.4 mm long. Leaves opposite, petiolate; petiole 4–13 mm, pubescent with erect golden trichomes 0.2 mm long, glabrescent; stipules deciduous, free, elliptic, 2.5–4 × 1–1.5 mm, pubescent with appressed golden trichomes, 0.3–0.5 mm long; lamina elliptic, 7–19 × 2.5–8 cm, adaxially pubescent on the mid-vein with appressed golden trichomes 0.1 mm long, glabrescent, abaxially pubescent with appressed golden trichomes 0.3–0.5 mm long, glabrescent, semi-craspedodromous, with 6–8 major secondary vein pairs, secondary vein spacing decreasing proximally between them, base symmetrical, cuneate, margin subcrenate, apex acuminate, acumen 0.5–1 cm, mucronate, leaf domatia absent. Inflorescence axillary or terminal, racemose, 3–10 cm, 1.5–3 cm diam., axis pubescent with erect golden trichomes 0.2 mm long; pedicels 3–6 mm, pubescent with erect golden trichomes 0.1–0.2 mm long, articulated at the base, peduncle bracts deciduous, triangular, 1.5–1.8 × 1.5–1.8 mm, herbaceous, pubescent with appressed golden trichomes 0.3–0.5 mm long, margin ciliolate, bractlets persistent, 1 × 1 mm, herbaceous, pubescent with appressed golden trichomes 0.1 mm long, margin ciliolate. Flowers 3–4 × 3–4 mm, sepals subequal in size and shape, triangular, 1.6–2 × 1–1.5 mm, 1–5-veined, pubescent on the distal part of the costa with appressed golden trichomes 0.1–0.2 mm long, margin ciliolate, apex apiculate; petals elliptic, 3–4 × 1.4–2 mm, costa pubescent with spreading golden trichomes 0.1 mm long, margin ciliolate, apex acute, white; stamens 2.5–3 mm long, all filaments united at the base forming a staminal tube, tube glabrous, filaments 0.4–0.5 mm, anthers elliptic, 1.1–1.4 × 0.4–0.6 mm, pubescent between the thecae adaxially with spreading golden trichomes 0.1–0.2 mm long, apex rounded, connective 0.7–0.8 mm, dorsal connective scale conspicuous, lanceolate, 2.3–2.5 × 0.8–1 mm, margin entire, orange-brown; ovary globose, 0.8–1 × 0.9–1 mm, pubescent with appressed golden trichomes 0.1–0.2 mm long; style filiform, curved, 1.5–2 × 0.1 mm, pubescent proximally with appressed trichomes 0.1 mm long, stigma acute. Fruit a symmetrical, subligneous capsule dehiscent along three sutures, ellipsoid, 1–2 × 0.5–0.8 cm, apex acuminate, tomentose with golden trichomes 0.1 mm long. Seeds one per valve, globose, 5–7 mm diam., glabrous, with maculae, brown when dry. (Fig. 10)

#### Distribution and habitat.

*Rinoreagaleanoae-bernalii* is apparently endemic to Colombia, where it occurs in the Departments of Antioquia, Caldas and Santander. Its distribution corresponds to the Biogeographical Province of Magdalena in the Pacific Dominion (*sensu*Morrone (2014)). It grows in lowland tropical rainforest at elevations of 192–500 m. (Fig. 9)

#### Etymology.

*Rinoreagaleanoae-bernalii* is named in honour of the late Dr. Gloria Galeano Garcés (1958–2016) and Dr. Rodrigo Bernal, a husband-wife botanical team who contributed greatly to the knowledge of the Colombian flora and the taxonomy of Neotropical palms, Cyclanthaceae and Marantaceae. As professors and mentors, they inspired many of their students to pursue a botanical career studying the flora of Colombia.

#### Phenology.

The species flowers between July and October; fruiting specimens were collected in January, April, July and September through November.

#### Conservation status.

*Rinoreagaleanoae-bernalii* has a geographic range in the form of an EOO of 9,409 km^2^ and AOO of 48 km^2^. It is known from 11 locations that are not included in any protected area. The locations in the middle Magdalena River Valley are heavily impacted by deforestation for grazing and agriculture, especially for cultivation of *Elaeisguineensis* (oil palm), while some known locations in Antioquia Department are threatened with limestone extraction for concrete production. Given ongoing habitat disturbance and projected continuing decline in the quality and extent of habitat, it is likely that the species will qualify in the near future for a threatened status and, thus, it is preliminarily assessed with a status of “Near Threatened” (NT).

#### Notes.

Hekking noted the unusual glabrous lamina in at least one specimen which he had identified as *Rinoreahirsuta* (e.g. *E. Rentería et al. 1779*). Based on the discrete biogeographic distributions of *R.galeanoae-bernalii* and *R.hirsuta* (Magdalena Valley vs. Chocó-Darien, respectively) and the morphological characters that differentiate the two (e.g. the presence of a staminal tube and the patterns of pubescence on the anther connectives and style), we hypothesise that *R.galeanoae-bernalii* is a separately evolving lineage worthy of recognition at the rank of species.

*Rinoreagaleanoae-bernalii* is sympatric with *R.cogolloi*, but it can be differentiated by the racemose inflorescence and glabrous seeds (vs. thyrsoid inflorescence and pilose seeds in *R.cogolloi*). *Rinoreagaleanoae-bernalii* is also sympatric with R.pubifloravar.pubiflora, but it can be differentiated by the valves that each contain one glabrous seed (vs. valves that each contain three pubescent seeds in R.pubifloravar.pubiflora); see Table 2 for a comparison of key diagnostic characters differentiating *R.galeanoae-bernalii*, *R.cogolloi* and R.pubifloravar.pubiflora.

#### Additional specimens examined.

**Colombia. Dept. Antioquia.** Mpio. Puerto Triunfo: corregimiento Puerto Perales, hacienda Cerritos, 7.65833, -74.81861, 192 m elev., Nov 2009 (fr), *L. Londoño et al. 938* (HUA). Mpio de Sonsón: Corregimiento de Jerusalén, Vía Medellín-Bogotá, predios de Sumicol, cuenca del Río Claro, 5.915969. -74.850459, 370 m elev., 16 Mar 2021 (fl), *J. M. Vélez et al. 7621* (MEDEL, HUA). Mpio de Yondó: ciénaga de Barbacoas, Hacienda Java, bosque Catanga, 6°43'N, 74°19'W, 100–130 m elev., 18 Apr 2011 (fr), *J. Betancur 15261 et al.* (COL, HUA, NYBG, US). **Dept. Caldas.** Mpio. La Dorada: vereda la Atarraya, finca los Achiles, relictos de bosque de la finca, 5°41'03.9"N, 74°44'16.4"W, 30 Jan 2021 (fr), *D. Sanín et al. 7704* (HUA, FAUC). Mpio. La Dorada: Vda. La Habana, 250 m elev., 16 Jan 2000 (fr), *M. V. Bustos Giraldo 62* (COL); predio el palmar, 5°18.551'N, 74°47.839'W, 305 m elev., 4 Nov 2012 (fl, fr), *J. M. Vélez 4303* (MEDEL). **Dept. Santander.** Mpio. Cimitarra: Potrero Quito, 6°28.285'N, 74°21.116'W, 3 Mar 1999 (st), *Á. Idárraga et al. 902* (HUA); corregimiento Puerto Olaya, Hacienda el Bosque, Puerto Arturo, 6°27.576'N, 74°21.034'W, 4 Aug 1999 (fl), *Á. Idárraga et al. 1491* (JAUM); corregimiento Puerto Olaya, Hacienda Piamonte, 6°26'17.8"N, 74°22'07.6"W, 29 May 2015 (fr), *Á. Idárraga et al. 6034* (HUA); *ibid*., 6°26'25.3"N, 74°22'22"W, 123 m elev., 22 Jul 2015 (fl), *Á. Idárraga et al. 6116* (HUA); *ibid*., 23 Jul 2015 (fl, fr) *Á. Idárraga et al. 6138* (HUA); Puerto Araujo, 500 m elev., 18 Sep 1979 (fr), *E. Rentería et al. 1779* (COL, HUA, JAUM, US); *ibid.*, 700 m elev., 20 Sep 1979 (fr), *E. Rentería et al. 1809* (COL); corregimiento Puerto Olaya, Hacienda el Bosque, fragmento Monte Cristo, 6°28'N, 74°21'W, 19 Sep 2001 (fl), *A. Rivas et al. 152* (HUA, JAUM); vereda los Ranchos, Hacienda Monterrey, 6°15'42"N, 74°27'51"W, 123 m elev., 1 Oct 1998 (fl, fr), *W. Rodríguez et al. 1070* (JAUM); *ibid*. (fl, fr), *W. Rodríguez et al. 1505* (JAUM). Mpio. Girón: vereda Sogamoso, hacienda Trigueros, 7°05'27.1"N, 73°21'41.4"W, 332 m elev., 25 Feb 2011 (fl, fr), *E. Y. Rodríguez-Ch. 1989* (COL).

### ﻿7. Rinorea betancurii

Taxon classificationPlantaeMalpighialesViolaceae

Hoyos-Gómez sp. nov.

556E7A97-2D6C-5F6E-B6E0-BB869FA37692

urn:lsid:ipni.org:names:77342877-1

#### Type.

Colombia. Dept. Caquetá: Mpio. de Solano, región de Araracuara, sector Chiribiquete, camino a Tepuí, 0°12'16"S, 72°29'14"W, 170 m elev., 10 Dec 2010, *F. Castro 10919* (holotype: COAH [acc. 78339]!; isotype: NY [barcode 02691382]!).

#### Description.

*Rinoreabetancurii* is similar to *R.macrocarpa* by the elliptic lamina lacking domatia, lamina base symmetrical and capsule symmetrical with apex acuminate, but it differs by the abaxial lamina surface pubescent (vs. abaxial lamina surface glabrous in *R.macrocarpa*), lamina base obtuse (vs. lamina base cuneate), fruit smaller 2.5–3 cm long (vs. fruit larger, 3–5.7 cm) and valves containing two pubescent seeds (vs. valves containing three glabrous seeds).

**Figure 11.**
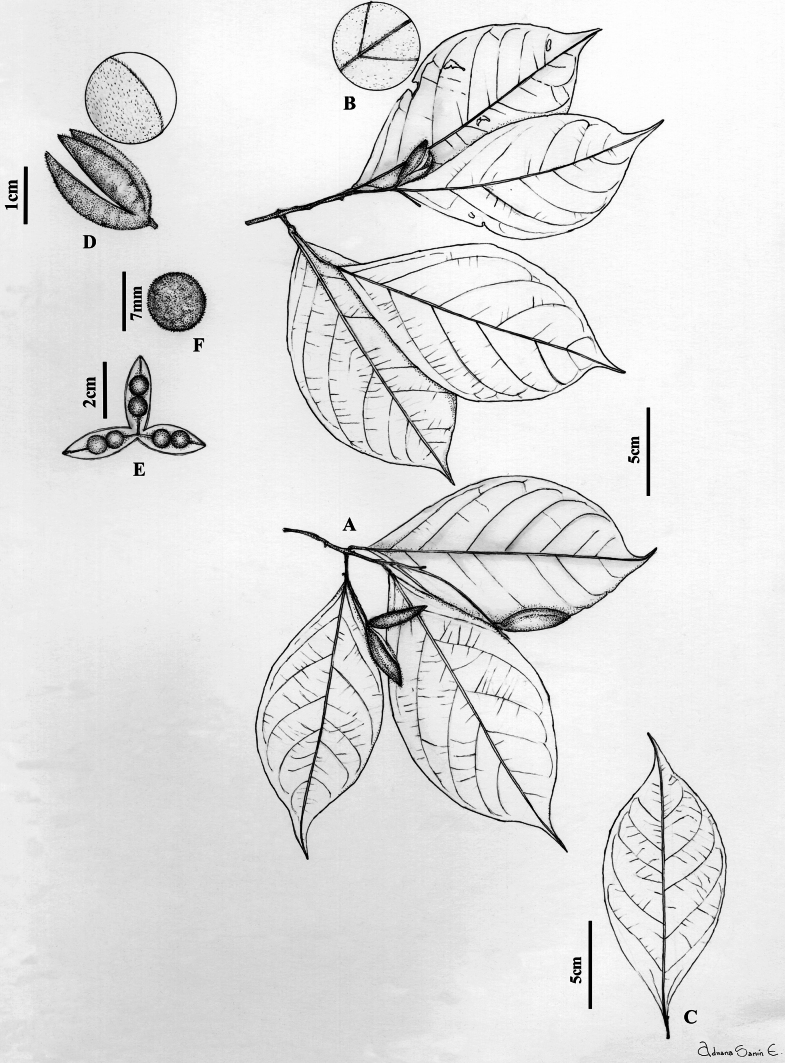
*Rinoreabetancurii* Hoyos-Gómez **A** habit **B** detail of leaf, abaxial surface **C** leaf architecture **D** fruit and detail of pubescence **E** detail of fruit showing two seeds per valve **F** seed. (**A–F**: *R. L. Liesner 15689* [MO]).

Treelets 1.5–5 m tall, branchlets with puberulent erect ferruginous trichomes 0.1–0.2 mm long, glabrescent. Leaves opposite, petiolate; petiole 2–4 mm long, pubescent with erect ferruginous trichomes 0.2 mm long; stipules deciduous, free, lanceolate, 2.3–3 × 1–1.1 mm, herbaceous, pubescent with appressed ferruginous trichomes 0.2 mm long, lamina elliptic, 6.3–14.4 × 2.6–7 cm, adaxially glabrous, abaxially pubescent with erect ferruginous trichomes 0.2–0.4 mm long, semi-craspedodromous, with 5–7 major secondary vein pairs, secondary veins with spacing between them decreasing proximally, symmetrical, base obtuse, margin entire to subcrenate, apex acute, acumen 8–14 mm long, mucronate, domatia absent. Flowers unknown. Fruit a symmetrical, subligneous capsule dehiscent along three sutures, ellipsoid, 2.5–3 × 0.5–0.6 cm, apex acuminate, pubescent with curved golden trichomes 0.3–0.4 mm long, green in vivo, brown when dry. Seeds two per valve, globose, 7–8 mm diam., pubescent with spreading trichomes 0.1–0.2 mm long, with maculae, seeds brown when dry. (Fig. 11)

#### Distribution and habitat.

*Rinoreabetancurii* occurs in Brazil, Colombia and Venezuela, an area that corresponds to the Biogeographic Provinces of Imerí and Pantepui in the Boreal Brazilian Dominion (*sensu*Morrone (2014)). The species grows in lowland tropical rainforest at elevations of 60–170 m. *Rinoreabetancurii* has been recorded as growing in sandy soils near rivers and on slopes and hills. (Fig. 4)

#### Etymology.

*Rinoreabetancurii* honours Julio Betancur, Colombian botanist and curator at the National Herbarium in Bogotá. Dr. Betancur has inspired a new generation of students to pursue a career in botany and has made many contributions to the taxonomic knowledge of the Bromeliaceae, Heliconiaceae and the flora of Colombia.

#### Phenology.

Fruiting specimens were collected in February through May and in September, November and December.

#### Conservation status.

*Rinoreabetancurii* has a geographic range in the form of an estimated EOO of 511,480 km^2^ and an AOO of 36 km^2^. The species is known from eight localities, none of which appear to be in protected areas. Based on satellite imagery from Google Earth, the locations in Colombia and Brazil appear to be heavily impacted by deforestation, while the four Venezuelan locations are in remote and vast primary forests with large buffers from disturbed areas. Even though we project continuing decline in the extent and quality of habitat for the Colombian and Brazilian locations, the undisturbed forests in southern Venezuela appear to be safe from large-scale disturbance for the foreseeable future. Nevertheless, it appears unlikely that the species will qualify for a threatened status in the near future and is assigned a preliminary status of Least Concern (LC).

#### Notes.

*Rinoreabetancurii* occurs with R.pubifloravar.pubiflora, but it can be separated by the valves that contain two seeds (vs. valves that contain three seeds in R.pubifloravar.pubiflora).

#### Additional specimens examined.

**Colombia. Dept. Caquetá**: cabeceras del río Masay, desembocadura del río San Jorge al río Cuñaré, 1–6 Mar 1980 (fr), *M. Pabón 946* (COL). **Dept. Meta**: Mpio. Puerto López, vereda el Tigre, finca Pista, orilla del canal, 8 Feb 2008 (fr), *F. Castro 4760* (UBDC). **Dept. Vaupés**: Mpio Taraira, comunidad Jotabeyá, 0°35'S, 70°11'W, 150–250 m elev., 27 Mar 2009 (fr), *J. Betancur et al. 13854* (COAH, COL, HUA). **Venezuela. Bolivar State**: Mpio. Foráneo Aripao, margen derecha del caño Minchaquene (Hormiga), tributario del Alto Caura, entre Araguaña y Campamento, 4°45'N, 64°12'W, 2 May 1988–5 May 1988 (fr), *G. Aymard 6810* (MO, NY, U). **Amazonas State**: Mpio. Atabapo, río Cunucunuma, raudal Mapaco, 3°37'N, 65°51'W, Apr 1990 (fr), *W. R. Anderson 13343* (F, MICH); close to cerro Neblina base camp on río Mawarinuma, 0°50'N, 66°10'W, 6 Feb 1984 (fr), *R. L. Liesner 15689* (BHO, MO, U, VEN); Cerro Yapacana, 3°45'N, 66°45'W, 125–400 m elev., 3 May 1970 (fr), *J. Steyermark et al. 113015* (COL). **Brazil. Amazonas State**: Mpio. Barcelos, base cerro Aracá, 0°42'N, 63°22'W, 14 Jul 1985 (fr), *I. Cordeiro 193* (INPA, NY).

### ﻿Key to species of Rinoreasect.Pubiflorae in Colombia

**Table:** 

1	Inflorescence racemose	**2**
–	Inflorescence thyrsoid	**22**
2	Lamina papery, base slightly asymmetric and oblique	**3**
–	Lamina membranous, papery or coriaceous, base symmetric and equilateral	**4**
3	Inflorescence 4–23 cm long; sepals ca. half as long as the petals; seeds two per valve, with maculae	***Rinoreadasyadena* A.Robyns**
–	Inflorescence 1–4 cm long; sepals nearly as long as the petals; seeds three per valve, without maculae	***Rinoreasylvatica* (Seem.) Kuntze**
4	Lamina coriaceous	**5**
–	Lamina papery or membranous	**7**
5	Lamina margin entire; style pilosulous at the base; seeds three per valve	***Rinoreamarginata* (Triana & Planch.) Rusby ex Johnston**
–	Lamina margin subcrenate to serrate; style strigose at the base, seeds two per valve	**6**
6	Lamina elliptic, 6.5–17 × 2.7–6 cm, margin subcrenate to subserrate	***Rinoreamelanodonta* S.F.Blake**
–	Lamina narrowly ovate, 11.5–21 × 4–7.5 cm, margin serrate	***Rinoreabrachythrix* S.F.Blake**
7	Indument composed of one type of trichome	**8**
–	Indument composed of two types of trichomes	**17**
8	Lamina lanceolate	***Rinoreaaymardii* Hoyos-Gómez**
–	Lamina elliptic or obovate to ovate	**9**
9	Lamina obovate to ovate, domatia absent	***Rinoreaovalifolia* (Britton) S.F.Blake**
–	Lamina elliptic, domatia present or absent	**10**
10	Domatia present	**11**
–	Domatia absent	**13**
11	Lamina margin ciliate	***Rinoreastevensii* Hoyos-Gómez**
–	Lamina margin not ciliate	**12**
12	Lamina pubescent on both surfaces; inflorescence 1.5–5 cm long; dorsal gland completely covering the filament	***Rinoreachiribiquetensis* Hoyos-Gómez**
–	Lamina glabrous on both surfaces; inflorescence 8–12.5 cm long; dorsal gland free, adnate to the filaments	***Rinoreafalcata* (Mart. ex Eichler) Kuntze**
13	Lamina abaxially pubescent, with trichomes on the entire surface	**14**
–	Lamina abaxially glabrous or glabrescent, if glabrescent, then trichomes only on the veins	**15**
14	Lamina with 9–14 lateral veins; seeds one per valve; glabrous; NW Colombia and Darién (Panama)	***Rinoreahirsuta* Hekking**
–	Lamina with 5–7 lateral veins; seeds two per valve, pubescent; Amazon Basin (Brazil, Colombia and Venezuela)	***Rinoreabetancurii* Hoyos-Gómez**
15	Filaments free, not forming a glandular tube; seeds two per valve, glabrous	***Rinoreaflavescens* (Aubl.) Kuntze**
–	Filaments fused forming a glandular tube; seeds one or three per valve	**16**
16	Pedicels 3–6 mm long; connective scales with entire margins; capsule pubescent, seeds one per valve; endemic to the Magdalena Valley, Colombia	***Rinoreagaleanoae-bernalii* Hoyos-Gómez**
–	Pedicels 0.5–1 mm long; connective scale with the margin proximally fringed; capsule sparsely ferruginous hispidulous; seeds three per valve	***Rinoreamacrocarpa* (Mart. ex Eichler) Kuntze**
17	Fruits with one seed per valve, seed glabrous	**18**
–	Fruits with two or three seeds per valve, seeds glabrous or pubescent	**19**
18	Lamina with 11–14 pairs of lateral veins; style golden pilosulous to strigillose proximally; Central America and Magdalena River Valley and Chocó Region in Colombia	***Rinoreasquamata* S.F.Blake**
–	Lamina with 7–10 pairs of lateral veins; style pilose proximally; Magdalena Valley and Amazon Basin (Bolivia, Brazil, Ecuador, Colombia, Peru)	***Rinoreaviridifolia* Rusby**
19	Fruits with three seeds per valve	**Rinoreapubifloravar.pubiflora**
–	Fruits with two seeds per valve	**20**
20	Seeds pilosulous, with maculae	**Rinoreapubifloraf.andersonii (Sandwith ex Hekking) Hekking**
–	Seeds glabrous, without maculae	**21**
21	Lamina with 8–10 pairs of lateral veins, margin subserrate; pedicels articulated near the base; dorsal gland pilose, seeds with maculae; Panamá and Chocó Region in Colombia	***Rinoreacallejasii* Hoyos-Gómez**
–	Lamina with 6–8 pairs of lateral veins, margin crenate; pedicels articulated near the middle; dorsal gland pubescent, seeds without maculae; Amazon Basin (Brazil, Colombia, Peru, Ecuador)	**Rinoreapubifloravar.grandifolia (Eichler) Hekking**
22	Lamina glabrous on upper surface, base symmetric, cuspidate at the apex, domatia absent; seeds one per valve, glabrous	***Rinoreavillosiflora* Hekking**
–	Lamina glabrous or pubescent on upper surface, base symmetric or asymmetric, apex always acuminate, domatia present or absent, seeds one or two per valve, glabrous or pubescent	**23**
23	Lamina base asymmetric, domatia absent; seeds one per valve	**24**
–	Lamina base symmetric, domatia present or absent; seeds one or two per valve	**26**
24	Seeds obovoid, glabrous, without maculae	***Rinoreaulmifolia* Kuntze**
–	Seeds globose, pubescent, with maculae	**25**
25	Lamina glabrous on upper surface; dorsal gland adnate to the filament, glabrous; capsule symmetric	***Rinorealindeniana* (Tul.) Kuntze**
–	Lamina pubescent on upper surface along the mid-vein; dorsal gland covering the filament, pubescent-pilose; capsule asymmetric	***Rinoreacogolloi* Hoyos-Gómez**
26	Domatia absent; capsule asymmetric or symmetric; seeds one or two per valve, glabrous or pubescent	**27**
–	Domatia present; capsule asymmetric; seeds two per valve, glabrous	**28**
27	Inflorescence 8–18 cm long; capsule asymmetric; seeds one per valve, glabrous	***Rinoreavaupesana* L.B.Sm. & Á.Fernández**
–	Inflorescences 1–8 cm long, capsule symmetric; seeds two per valve, pubescent	***Rinoreahummelii* Sprague**
28	Petals elliptic to ovate, 1.7–2.2 mm long, pilose along the costa; stamens 1.2–1.7 mm long, glandular tube glabrous; seeds one per valve	***Rinorearacemosa* (Mart.) Kuntze**
–	Petals narrowly ovate, 3–3.5 mm long, pubescent along the costa; stamens 2.2–3 mm long, glandular tube ciliate; seeds two per valve	***Rinoreasprucei* (Eichler) Kuntze**

## Supplementary Material

XML Treatment for Rinorea callejasii

XML Treatment for Rinorea aymardii

XML Treatment for Rinorea chiribiquetensis

XML Treatment for Rinorea stevensii

XML Treatment for Rinorea cogolloi

XML Treatment for Rinorea galeanoae-bernalii

XML Treatment for Rinorea betancurii

## ﻿Additional information

### Author ORCIDs

Saúl E. Hoyos-Gómez https://orcid.org/0000-0001-7068-6667

Ricardo Callejas Posada https://orcid.org/0000-0001-6333-5682

Gregory A. Wahlert https://orcid.org/0000-0003-1703-9860

### Data availability

All of the data that support the findings of this study are available in the main text.

## References

[B1] Aymard G (2000) Estudio de la composición florística en bosques de *terra firme* del alto río Orinoco, estado Amazonas, Venezuela. Acta Botanica Venezuelica 23: 123–156.

[B2] Bachman SJ Moat AW Hill J De la Torre Scott B (2011) Supporting Red List threat assessments with GeoCAT: Geospatial conservation assessment tool. ZooKeys 150: 117–126. 10.3897/zookeys.150.2109 PMC3234434 22207809

[B3] Ballard Jr HE Paula-Souza JD Wahlert GA (2014) Violaceae. In: Kubitzki K (Ed.) The Families and Genera of Vascular Plants.Springer Verlag, Berlin, 303–322. 10.1007/978-3-642-39417-1_25

[B4] De Queiroz K (2008) Species concepts and species delimitation. Systematic Biology 56(6): 879–886. 10.1080/10635150701701083 18027281

[B5] Ellis B Daly D Hickey L Johnson K Mitchell J Wilf P Wing S (2009) Manual of Leaf Architecture. Cornell University Press, Ithaca, 190 pp. 10.1079/9781845935849.0000

[B6] Hans ter Steege H Pitman NCA Sabatier D Baraloto C Salomão RP Ernesto Guevara J Phillips OL Castilho CV Magnusson WE Molino J-F Monteagudo A Núñez Vargas P Carlos Montero J Feldpausch TR Honorio Coronado EN Killeen TJ Mostacedo B Vasquez R Assis RL Terborgh J Wittmann F Andrade A Laurance WF Laurance SGW Marimon BS Marimon Jr B-H Guimarães Vieira IC Leão Amaral I Brienen R Castellanos H Cárdenas López D Duivenvoorden JF Mogollón HF de Almeida Matos FD Dávila N García-Villacorta R Stevenson Diaz PR Costa F Emilio T Levis C Schietti J Souza P Alonso A Dallmeier F Duque Montoya AJ Fernandez Piedade MT Araujo-Murakami A Arroyo L Gribel R Fine PVA Peres CA Toledo M Aymard GA Baker TR Cerón C Engel J Henkel TW Maas P Petronelli P Stropp J Zartman CE Daly D Neill D Silveira M Ríos Paredes M Chave J de Andrade Lima Filho D Jørgensen PM Fuentes A Schöngart J Valverde FC Di Fiore A Jimenez EM Peñuela Mora MC Fernando Phillips J Rivas G van Andel TR von Hildebrand P Hoffman B Zent EL Malhi Y Prieto A Rudas A Ruschell AR Silva N Vos V Zent S Oliveira AA Cano Schutz A Gonzales T Trindade Nascimento M Ramirez-Angulo H Sierra R Tirado M Umaña Medina MN van der Heijden G Vela CIA Vilanova Torre E Vriesendorp C Wang O Young KR Baider C Balslev H Ferreira C Mesones I Torres-Lezama A Urrego Giraldo LE Zagt R Alexiades MN Hernandez L Huamantupa-Chuquimaco I Milliken W Palacios Cuenca W Pauletto D Valderrama Sandoval E Valenzuela Gamarra L Dexter KG Feeley K Lopez-Gonzalez G Silman MR (2013) Hyperdominance in the Amazonian Tree Flora. Science 342(6156): 1243092. 10.1126/science.1243092 24136971

[B7] Hekking WHA (1988) Violaceae. Part I–Rinorea and Rinoreocarpus. Flora Neotropica, Monograph 46. The New York Botanical Garden, New York, 207 pp.

[B8] IUCN (2012) IUCN Red List Categories and Criteria: version 3.1. Second edition. IUCN, Gland, Switzerland and Cambridge, UK, 2 pp. http://www.iucnredlist.org/technical-documents/categories-and-criteria [Last accessed January 2023]

[B9] Morrone JJ (2014) Biogeographical regionalisation of the Neotropical region. Zootaxa 3782: 01–110. 10.11646/zootaxa.3782.1.1 24871951

[B10] Oliveira JFC Paganucci de Queiroz L (2020) *Rinoreagemmulata* (Violaceae): A new species from eastern Brazil. Phytotaxa 435: 050–056. 10.11646/phytotaxa.435.1.6

[B11] Silva N Medeiros E (2012) Uma Nova Espécie de Rinorea Aubl. (Violaceae) do Brasil. Revista de Biologia Neotropical. Goiânia 9(1): 1–3. 10.5216/rbn.v9i1.12622

[B12] Thiers B (2023) [continuously updated] Index Herbariorum: A global directory of public herbaria and associated staff. New York Botanical Garden’s Virtual Herbarium. http://sweetgum.nybg.org/science/ih/ [Last accessed January 2023]

[B13] van Velzen R Wahlert GA Sosef MSM Onstein RE Bakker FT (2015) Phylogenetics of *Rinorea* (Violaceae): Elucidating infrageneric relationships using plastid and nuclear DNA sequences. Systematic Botany 40(1): 174–184. 10.1600/036364415X686486

[B14] Wahlert GA Ballard Jr HE (2009) A new zygomorphic-flowered *Rinorea* (Violaceae) from the Neotropics. Novon 19(3): 416–420. 10.3417/2007155

[B15] Wahlert GA (2010) Phylogeny, biogeography, and a taxonomic revision of *Rinorea* (Violaceae) from Madagascar and the Comoro Islands. PhD Thesis, Ohio University, 324 pp.

[B16] Wahlert GA Ballard Jr HE (2012) A phylogeny of *Rinorea* (Violaceae) inferred from plastid DNA sequences with an emphasis on the African and Malagasy species. Systematic Botany 37(4): 964–973. 10.1600/036364412X656392

[B17] Wahlert GA Hoyos–Goméz SE Ballard Jr HE (2017) Systematic studies in Neotropical *Rinorea* (Violaceae): Two new sections and a new generic segregate. Brittonia 70(1): 140–147. 10.1007/s12228-017-9507-z

